# Brain and Liver Dual‐Targeting Oridonin Nanoparticles to Enhance Aβ Clearance for Alzheimer's Disease Therapy

**DOI:** 10.1002/advs.202523458

**Published:** 2026-04-07

**Authors:** Wenshuai Gong, Wenting Hui, Sai Qiao, Qifeng Ji, Miao Liu, Bangle Zhang, Daozhou Liu, Yumei Wu, Siyuan Zhou

**Affiliations:** ^1^ Department of Pharmaceutics School of Pharmacy Air Force Medical University Xi'an China; ^2^ Department of Pharmacology School of Pharmacy Air Force Medical University Xi'an China

**Keywords:** Alzheimer's disease, apoferritin, brain and liver dual‐targeting, multifunctional nanoparticle, oridonin

## Abstract

The brain and liver are both critical organs involved in the pathogenesis of Alzheimer's disease (AD), particularly in the modulation of amyloid‐beta (Aβ) metabolism and neuroinflammation. Based on this, a multifunctional nanodrug delivery system, termed OAF, was developed by encapsulating oridonin (ORI) into apoferritin (ApoFn), enabling simultaneous targeting of both brain and the liver through transferrin receptor 1 (TfR1). OAF upregulated the expression of low‐density lipoprotein receptor‐related protein 1 (LRP1) in cerebral capillary endothelial cells and hepatic parenchymal cells to promote Aβ clearance from the brain and subsequent hepatic degradation. In AD mice, OAF treatment markedly reduced Aβ deposition, neuroinflammation, and cognitive impairment, while ameliorating inflammation, oxidative stress, and mitochondrial dysfunction in both brain and liver. Overall, OAF synergistically combined Aβ clearance, anti‐inflammatory, and antioxidant mechanisms, offering a novel therapeutic strategy for AD.

## Graphical abstract

We developed a nanoparticle named OAF, which simultaneously targeted to both the brain and liver via the transferrin receptor 1 (TfR1) receptor, promoting lipoprotein receptor‐related protein 1 (LRP1) expression to enhance amyloid‐beta (Aβ) clearance. In AD mice model, OAF significantly reduced Aβ deposition and cognitive impairment, while a mitigating inflammation and oxidative stress in liver and brain. This provided a novel synergistic therapeutic strategy for AD.

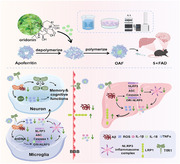

## 1 Introduction

Alzheimer's disease (AD) is one of the most common neurodegenerative diseases, impairing brain health and leading to severe neurological dysfunction and dyskinesia, which has been considered an incurable disease [1, 2, 3]. According to the World Alzheimer Report 2019, approximately 50 million people worldwide are currently living with dementia, and this number is projected to triple by 2050 [4]. Current treatments, including cholinesterase inhibitors and *N*‐methyl‐d‐aspartic acid receptor antagonists, provide only transient symptomatic relief without modifying disease progression [5]. Their diminishing efficacy underscores the critical need for therapies that target the root mechanisms of AD.

A central event in AD pathogenesis is the production and aggregation of amyloid‐beta (Aβ), which evolves into neurotoxic oligomers, fibrils, and ultimately cerebral amyloid plaques strongly correlated with disease severity [6, 7, 8]. Consequently, Aβ clearance has become a cornerstone of therapeutic development. Although three anti‐Aβ monoclonal antibodies, Aducanumab, Lecanemab, and Donanemab, have received FDA approval, their utilities are constrained by safety concerns such as Fc‐mediated inflammatory activation, cerebral edema, and microhemorrhages [9]. Moreover, these antibodies primarily focus on central pathology, while overlooking peripheral factors in the development of Alzheimer's disease.

Notably, recent studies indicated that approximately 40%–60% of brain‐derived Aβ is cleared in the periphery [10]. Growing evidence indicates that the liver plays a critical role in peripheral Aβ clearance and inflammatory regulation [11, 12, 13]. Clinical studies have observed that markers of liver dysfunction often emerge prior to AD diagnosis and correlate with plasma Aβ levels [14]. In APP/PS1 mice model, hepatomegaly, amyloid deposition, and oxidative stress occur even earlier than cerebral pathological changes, further supporting a proactive role of the liver in AD pathogenesis [15, 16]. Low‐density lipoprotein receptor‐related protein 1 (LRP1) serves as a pivotal regulator of Aβ clearance across both peripheral and central nervous system (CNS). In the liver, LRP1 mediates the endocytosis and lysosomal degradation of peripheral Aβ. Simultaneously, LRP1 expression on cerebral capillary endothelial cells facilitates Aβ efflux from the brain into the systemic circulation, enabling subsequent peripheral clearance [17, 18]. During AD progression, impaired hepatic function downregulates LRP1, which contributes to peripheral Aβ accumulation [19]. Systemic inflammation further suppresses the LRP1 expression on cerebral capillary endothelial cells, creating a vicious cycle of Aβ accumulation and neuroinflammation [20]. Chronic Aβ exposure amplifies neuroinflammation via the activation of microglial NLR family pyrin domain containing protein 3 (NLRP3) and upregulation of β‐secretase, while dysfunctional liver signaling exacerbates central inflammation via circulating cytokines [21]. Consequently, promoting the efflux of Aβ from the brain while strengthening peripheral Aβ clearance capacity may represent an effective strategy to alleviate AD pathology.

Oridonin (ORI), a natural diterpenoid from *Rabdosia rubescens*, exhibits potent anti‐inflammatory and neuroprotective properties, showing distinctive promise for AD treatment [22]. Unlike conventional symptom‐targeting agents, ORI directly inhibits NLRP3 inflammasome assembly via covalent binding to the cysteine 279, mitigating neuroinflammation [23]. It also avoids the adverse effects associated with Aβ immunotherapies, such as edema and microhemorrhages. Studies demonstrate that ORI improves synaptic integrity [24] and cognition in AD models, and attenuates systemic inflammation in liver injury models via the suppression of NLRP3 [25], suggesting its unique potential for dual‐organ protection. These properties position ORI as a highly promising candidate for AD therapy. However, its further clinical translation is currently limited by poor solubility, low oral bioavailability, short half‐life, and inadequate blood‐brain barrier (BBB) penetration [26]. Therefore, the development of an efficient delivery system capable of effectively loading and targeted delivery of ORI is imperative and urgent to overcome these challenges and fully exploit its therapeutic potential. However, existing nanoparticle‐based delivery systems often face several limitations including poor stability and biodegradability and difficulty in precise targeting tissue and cell type in vivo. A major concern for clinical translation of metal‐based nanoparticles is their potential toxicity [27, 28, 29, 30]. Collectively, these challenges impede their clinical translation.

Apoferritin (ApoFn) is a natural hollow protein cage nanostructure formed through the self‐assembly of 24 subunits, with an outer diameter of approximately 12 nm and an inner cavity of about 8 nm [31]. This self‐assembly process is highly controllable and reproducible, enabling the formation of structurally uniform nanocarriers under mild conditions. ApoFn exhibits excellent biocompatibility and low immunogenicity [32]. Its unique pH‐dependent disassembly behavior in acidic environments (e.g., lysosomes) allows controlled release of encapsulated drugs, thereby enhancing site‐specific delivery efficiency [33]. Furthermore, ApoFn offers advantages such as straightforward preparation, high drug‐loading capacity, and favorable stability. Its uniform nanoscale size helps to avoid rapid clearance by the reticuloendothelial system, prolonging it circulation time in vivo [31]. More importantly, ApoFn demonstrates efficient targeting capability toward both the liver and brain tissue. This is because transferrin receptor 1 (TfR1) is highly expressed in hepatocytes, brain capillary endothelial cells, microglia, and neurons. TfR1‐mediated endocytosis enhances the active targeting delivery of ApoFn [34, 35, 36].

**SCHEME 1:**
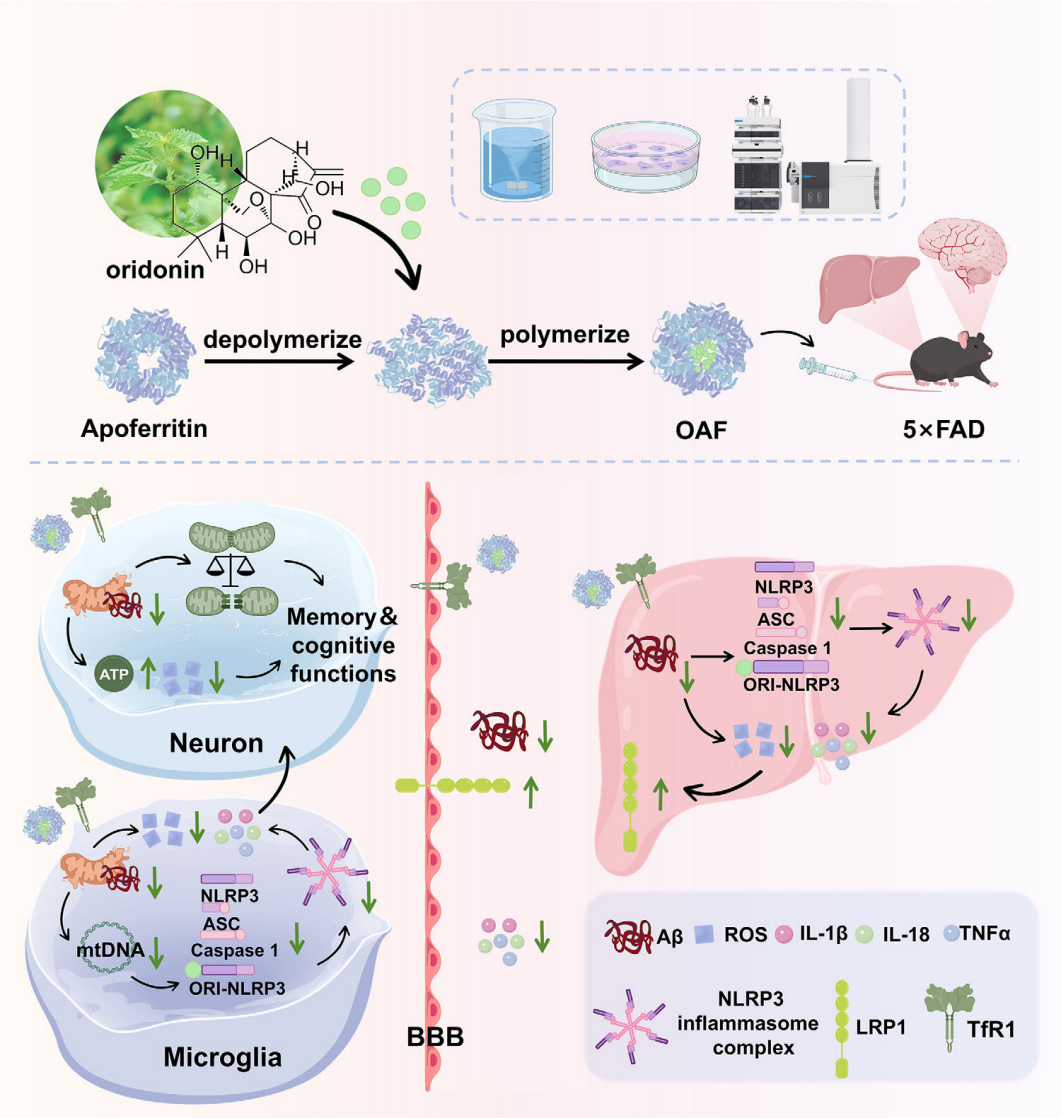
Schematic illustration of the preparation of OAF nanoparticles and their mechanism of action in treating Alzheimer's disease. The multifunctional nanoparticles target the brain and liver to synergistically clear Aβ, suppress neuroinflammation, and mitigate oxidative stress. Schematic was created with BioGDP.com [37].

Based on these properties, this study innovatively developed an ApoFn‐based nanoparticle loaded with ORI (OAF), aiming to improve the aqueous solubility and stability of ORI while achieving targeting delivery to both the liver and brain. This approach offers a novel strategy for combined central and peripheral treatment of AD through simultaneously targeting the brain and liver (Scheme 1).

## 2 Results and Discussion

### 2.1 Synthesis and Characterization of OAF Nanoparticles

We employed an encapsulation strategy based on the self‐assembly properties of ApoFn to prepare OAF. Specifically, 55% acetone was used to disrupt hydrogen bonds and hydrophobic interactions within the protein structure, leading to a loosened shell configuration of ApoFn with sufficient gaps or channels between subunits [38]. At this stage, ORI dissolved in acetone was introduced, allowing ORI molecules to diffuse into the hydrophobic inner cavity of ApoFn driven by the concentration gradient. Subsequent evaporation of acetone triggered the reassembly of the nanocage structure, restoring intersubunit interactions and encapsulating the ORI molecules within the cavity. The resulting product was purified by filtration through a 0.22 µm membrane and ultrafiltration centrifugation to completely remove unencapsulated free ORI, yielding pure OAF nanoparticles.

**FIGURE 1:**
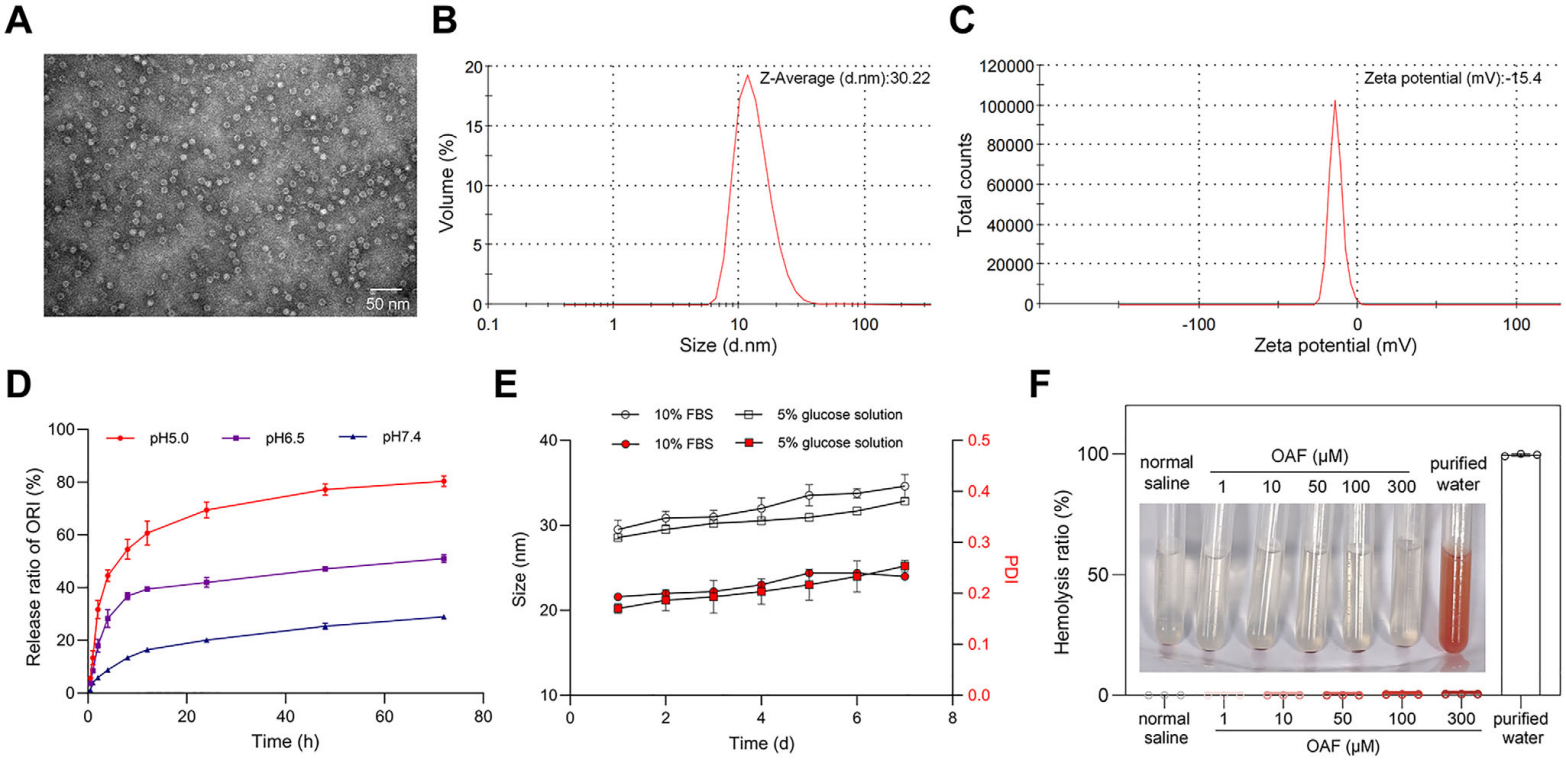
Characterization of OAF. (A) TEM image of OAF. (B) Particle size distribution of OAF. (C) Zeta potential distribution of OAF. (D) In vitro ORI release from OAF in different pH release medium. (E) Particle sizes and PDI of OAF in 5% w/w glucose solution or 10% v/v FBS solution. (F) Hemolysis effect of OAF. Data were presented as mean ± standard deviation (SD), *n* = 3.

Transmission electron microscopy (TEM) revealed that OAF exhibited a typical and well‐defined core–shell morphology, highly consistent with that of empty ApoFn, with uniform particle size distribution (Figure 1A and Figure S1), indicating the successful construction of OAF nanoparticles via the self‐assembly‐based encapsulation approach. Dynamic light scattering (DLS) further showed that the average hydrodynamic diameters of ApoFn and OAF were approximately 30 nm (Figure 1B and Figure S2A), with zeta potentials of −16.3 and −15.4 mV, respectively (Figure 1C and Figure S2B). No statistically significant differences were observed in either size or surface charge between ApoFn and OAF, confirming that the drug encapsulation process did not alter the structural integrity of ApoFn and OAF possessed favorable structural stability.

The drug release behavior of OAF was next evaluated under different pH conditions to verify its pH‐responsive characteristics. In pH 7.4 medium, over (87.1 ± 1.55) % of free ORI diffused from inside to outside the dialysis bag within 4 h when ORI was suspended in dialysis bag. However, only (8.79 ± 0.48) % of ORI was released from OAF to the outside of the dialysis bag within 4 h when OAF was suspended in dialysis bag (Figure S3). In pH5.0 medium, the cumulative release of ORI from OAF reached 70% within 24 h, significantly higher than that in pH6.0 and pH7.4 medium (Figure 1D). These results indicated that OAF underwent spontaneous disassembly under acidic lysosomal conditions while minimizing premature drug release before reaching target sites, thereby potentially enhancing the bioavailability of ORI.

Stability tests demonstrated that the particle size and zeta potential of OAF remained stable over 7 days in both 5% w/w glucose solution and 10% v/v fetal bovine serum (FBS) solution (Figure 1E and Figure S4), confirming not only its suitability for intravenous infusion but also its resistance to aggregation induced by serum protein adsorption. The storage stability of the lyophilized OAF was also evaluated. The formulation remained intact for 6 months at 4°C (Figure S5). Additionally, after the lyophilized OAF was redispersed in 5% glucose solution or 10% FBS solution, no significant changes in the particle size and zeta potential were observed within 7 days (Figure S6). These data confirmed that the OAF formulation remains stable for at least 6 months under the recommended storage conditions, providing strong support for its further translational development.

The drug loading capacity of OAF was measured via high‐performance liquid chromatography (HPLC), which was determined to be 30.45%, reflecting efficient encapsulation. Additionally, no hemolysis was observed at 300 µm OAF (Figure 1F), and OAF did not significantly affect the viability of primary neuronal cells at concentrations ranging from 2.5 to 30 µm (Figure S7), indicating compatibility with blood components and preliminary cellular safety.

### 2.2 OAF Facilitated the Clearance and Metabolism of Aβ_1–42_ in the Liver–Brain Microenvironment

**FIGURE 2:**
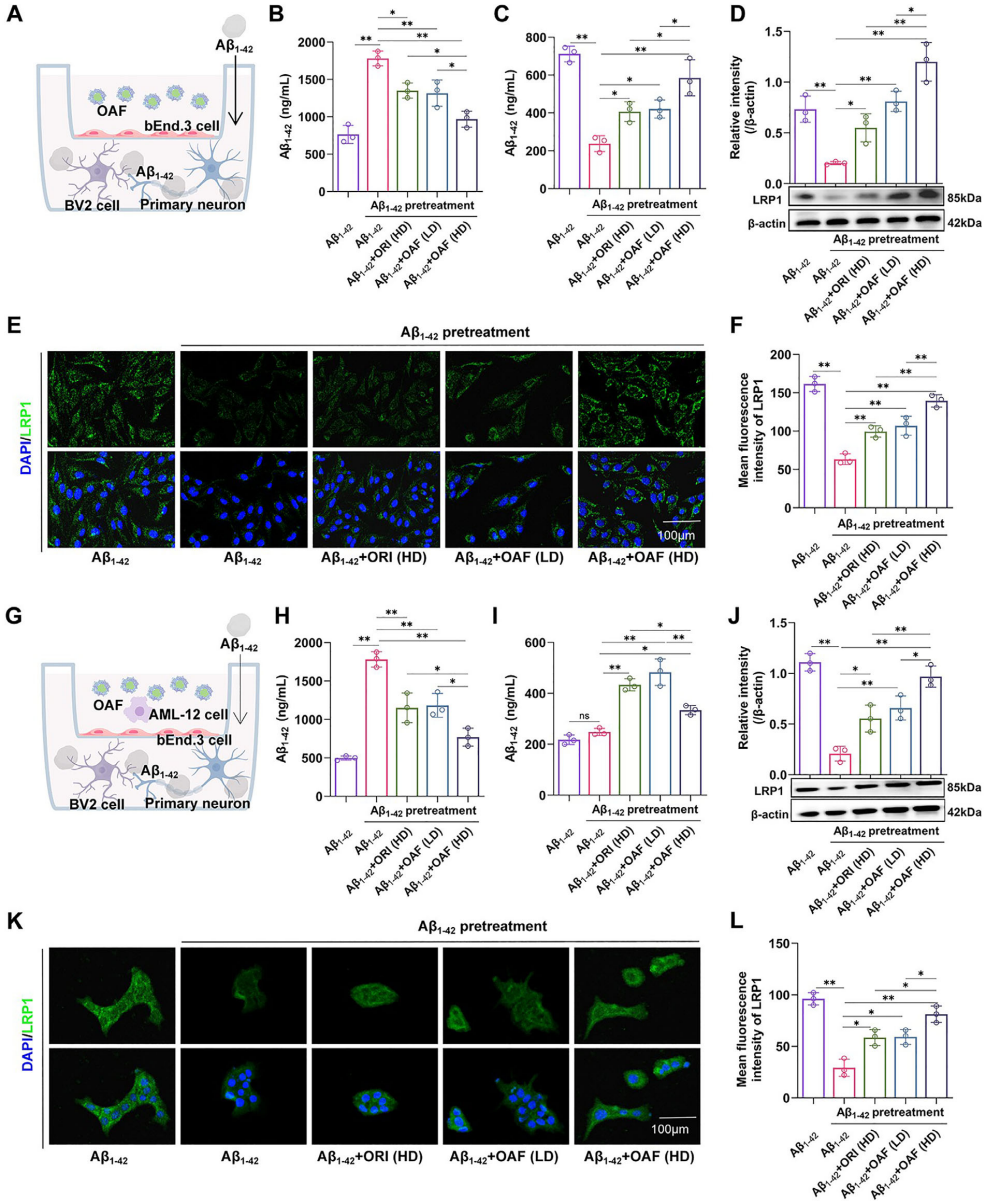
OAF enhanced the efflux and metabolism of Aβ_1_–_42_ in the liver–brain microenvironment. (A) Schematic diagram of the cerebral microenvironment model containing Aβ_1_–_42_. (B) The levels of soluble Aβ_1−42_ in recipient chamber medium in cerebral microenvironment model. (C) The levels of soluble Aβ_1−42_ in donor chamber medium in cerebral microenvironment model. (D) Western blot analysis of the effect of OAF on the expression of LRP1 in bEnd.3 cells in cerebral microenvironment model. (E,F) Immunofluorescence analysis of the effect of OAF on LRP1 expression in bEnd.3 cells in cerebral microenvironment model. (G) Schematic diagram of the liver–brain microenvironment model containing Aβ_1_–_42_. (H) The levels of soluble Aβ_1–42_ in recipient chamber medium in liver–brain microenvironment model. (I) The levels of soluble Aβ_1–42_ in donor chamber medium in liver–brain microenvironment model. (J) Western blot analysis of the effect of OAF on the expression of LRP1 in AML‐12 cells in liver–brain microenvironment model. (K,L) Immunofluorescence analysis of the effect of OAF on LRP1 expression in AML‐12 cells in liver–brain microenvironment model. HD: 10 µm OAF (molar equivalent of ORI). LD: 5 µm OAF (molar equivalent of ORI). Data were presented as mean ± SD, *n* = 3. ^*^p < 0.05, ^**^p < 0.01. Statistical significance was calculated via one‐way analysis of variance (ANOVA) with Tukey's test.

At first, the in vitro BBB model was built by using bEnd.3 cells. After the cells reached confluence and formed a monolayer, the transepithelial electrical resistance (TEER) between donor and recipient chamber was measured and found to stabilize at 200 Ω·cm^2^ approximately, indicating effective restriction of ion diffusion and mimicking the high‐resistance characteristics of the BBB in vivo [39, 40, 41]. Then, an in vitro cerebral microenvironment model was constructed by placing coverslips containing mature primary neurons and BV2 cells into recipient chamber below the established BBB monolayer. To simulate the pathological state of Aβ deposition in AD, Aβ_1_–_42_ was introduced into this in vitro model (Figure 2A). OAF significantly reduced Aβ_1_–_42_ level in the recipient chamber medium, while concurrently increasing its level in the donor chamber medium in a concentration‐dependent manner (Figure 2B,C). This indicated that OAF facilitated the efflux of Aβ_1_–_42_ across the BBB. Immunofluorescence and western blot analysis further revealed that in Aβ_1_–_42_‐containing cerebral microenvironment models, the expression of LRP1 in bEnd.3 cells was significantly downregulated compared with the normal group, while OAF increased the expression of LRP1 in bEnd.3 cells (Figure 2D‐F).

To evaluate the effect of OAF on the peripheral clearance of Aβ, a liver–brain microenvironment model was established in vitro. As illustrated in Figure 2G, liver–brain microenvironment model was developed by additionally introducing a coverslip with AML‐12 cells into the donor chamber. Both ORI and OAF promoted the transport of Aβ_1_–_42_ across the BBB from the receptor chamber into the donor chamber containing AML‐12 cells. Moreover, at equivalent ORI concentrations, the high‐dose OAF group exhibited significantly lower Aβ_1_–_42_ levels in the donor chamber medium than the free ORI group (Figure 2H,I). Mechanistically, immunofluorescence and western blot analysis demonstrated that Aβ_1_–_42_ decreased the LRP1 expression of AML‐12 cells in the liver–brain microenvironment model. Both ORI and OAF treatment restored LRP1 expression, with the high‐dose OAF group showing superior efficacy compared to the free ORI group (Figure 2J–L). It is noteworthy that, with other variables held constant, the high‐dose OAF group showed markedly less Aβ_1_–_42_ in the donor chamber in liver–brain microenvironment model compared to that in cerebral microenvironment model (Figure 2C,I).

In summary, ORI and OAF upregulated LRP1 expression in bEnd.3 and AML‐12 cells in liver–brain microenvironment model, thereby enhancing the efflux of Aβ_1_–_42_ from the recipient chamber and promoting its uptake and metabolism by liver cells in donor chamber. Furthermore, OAF demonstrated stronger effects than free ORI, highlighting the pharmacodynamic advantages of this nanodelivery system.

### 2.3 OAF Inhibited the Activation of NLRP3 Inflammasome by Alleviating Aβ_1_–_42_‐Induced Mitochondrial Damage

**FIGURE 3:**
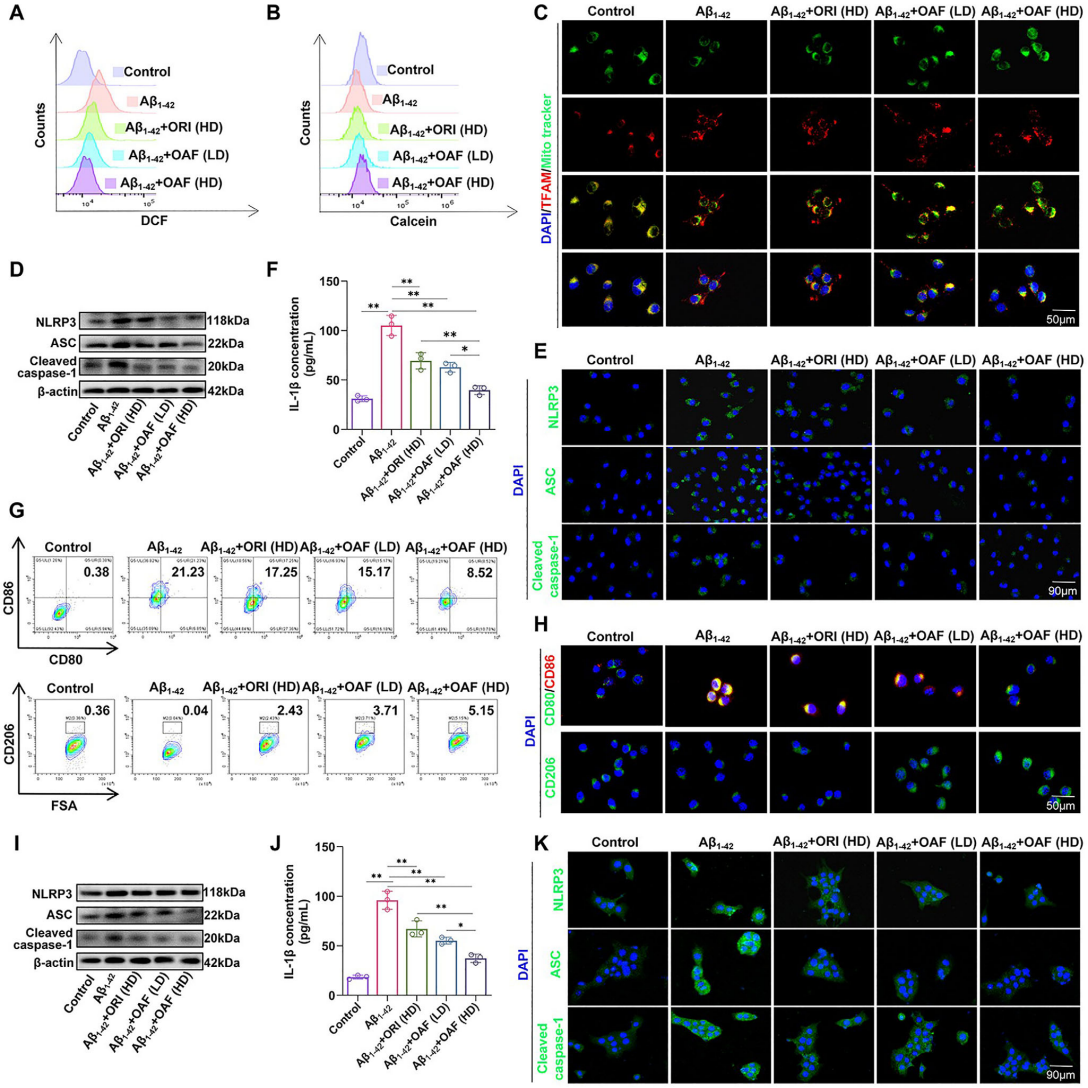
OAF inhibited the activation of NLRP3 inflammasome by alleviating Aβ_1_–_42_‐induced mitochondrial damage. (A) Flow cytometry analysis of the effect of ORI, OAF on the intracellular ROS level in BV2 cells in liver–brain microenvironment model. (B) Flow cytometry analysis of the effect of ORI, OAF on opening of mPTP in BV2 cells in liver–brain microenvironment model. (C) LSCM images of the released mitochondrial DNA (mtDNA) in the cytoplasm of BV2 cells in liver–brain microenvironment model. (D) Western blot analysis of the effect of ORI, OAF on the expression of NLRP3 inflammasome‐related proteins in BV2 cells in liver–brain microenvironment model. (E) LSCM images of the effect of ORI, OAF on the expression of NLRP3 inflammasome‐related proteins in BV2 cells in liver–brain microenvironment model. (F) IL‐1β levels in recipient chamber medium in liver–brain microenvironment model. (G) Flow cytometry analysis of M1‐ and M2‐BV2 cells in liver–brain microenvironment model. (H) LSCM images of M1‐ and M2‐BV2 cells in liver–brain microenvironment model. (I) Western blot analysis of the effect of ORI, OAF on the expression of NLRP3 inflammasome‐related proteins in AML‐12 cells in liver–brain microenvironment model. (J) IL‐1β levels in donor chamber medium in liver–brain microenvironment model. (K) LSCM images of the effect of ORI, OAF on the expression of NLRP3 inflammasome‐related proteins in AML‐12 cells in liver–brain microenvironment model. HD: 10 µm OAF (molar equivalent of ORI). LD: 5 µm OAF (molar equivalent of ORI). Data were presented as mean ± SD, *n* = 3. ^*^p < 0.05, ^**^p < 0.01. Statistical significance was calculated via one‐way ANOVA with Tukey's test.

Mitochondrial dysfunction plays a central role in AD development [42, 43]. Elevated levels of Aβ induce the production of reactive oxygen species (ROS), which in turn leads to mitochondrial damage [44]. In the liver–brain microenvironment model, Aβ_1_–_42_ significantly increased ROS levels in BV2 cells, induced abnormal opening of the mitochondrial permeability transition pore (mPTP), and promoted the release of mitochondrial DNA (mtDNA) into the cytoplasm (Figure 3A–C and Figures S8 and S9). Further functional assays confirmed that Aβ_1_–_42_ caused severe mitochondrial impairment, as evidenced by a significant decrease in the cellular oxygen consumption rate (OCR), reduced mitochondrial membrane potential, and downregulated expression of the key mitochondrial biogenesis protein PGC‐1α (Figure S10–S12). In contrast, OAF treatment not only effectively inhibited ROS generation, stabilized mPTP, and reduced mtDNA leakage but also significantly enhanced OCR, restored mitochondrial membrane potential, and upregulated PGC‐1α expression in BV2 cells liver–brain microenvironment model.

Cytosolic mtDNA can bind to NLRP3 and activate the inflammasome, promoting the assembly of NLRP3, ASC, and caspase‐1 into an oligomeric protein complex [45, 46]. Western blot and immunofluorescence analysis showed that Aβ_1_–_42_ stimulation markedly upregulated the expression of NLRP3, ASC, and cleaved caspase‐1 in BV2 cells in liver–brain microenvironment model, whereas OAF treatment significantly downregulated the expression of these key inflammatory proteins (Figure 3D,E and Figure S13). Enzyme‐linked immunosorbent assay (ELISA) further confirmed that Aβ_1_–_42_ increased the level of IL‐1β, IL‐18, and TNF‐α in liver–brain microenvironment model, and OAF effectively suppressed IL‐1β, IL‐18, and TNF‐α secretion (Figure 3F and Figure S14). These results indicated that OAF interrupted the activation of the mtDNA–NLRP3 inflammatory signaling axis.

At the phenotypic level, Aβ_1_–_42_ stimulation‐induced polarization of BV2 cells toward a proinflammatory M1 phenotype, characterized by enlarged cell bodies, blurred contours, and an amoeboid‐like morphology (Figure S15). Flow cytometry and immunofluorescence analysis also revealed an increased proportion of M1‐polarized cells accompanied by a decrease in the anti‐inflammatory M2 phenotype. OAF treatment significantly reduced the proportion of M1 phenotype while increasing the proportion of M2 phenotype (Figure 3G,H and Figure S16). Moreover, Aβ_1_–_42_ also triggered a pronounced inflammatory response in AML‐12 cells in liver–brain microenvironment model, and OAF was able to effectively attenuate inflammatory signaling in this system as well (Figure 3I–K and Figure S17). In all above experiment, the efficacy of OAF was significantly superior to that of free ORI.

### 2.4 OAF Alleviated Mitochondrial Damage and Apoptosis of Neurons in the Liver–Brain Inflammatory Microenvironment

**FIGURE 4:**
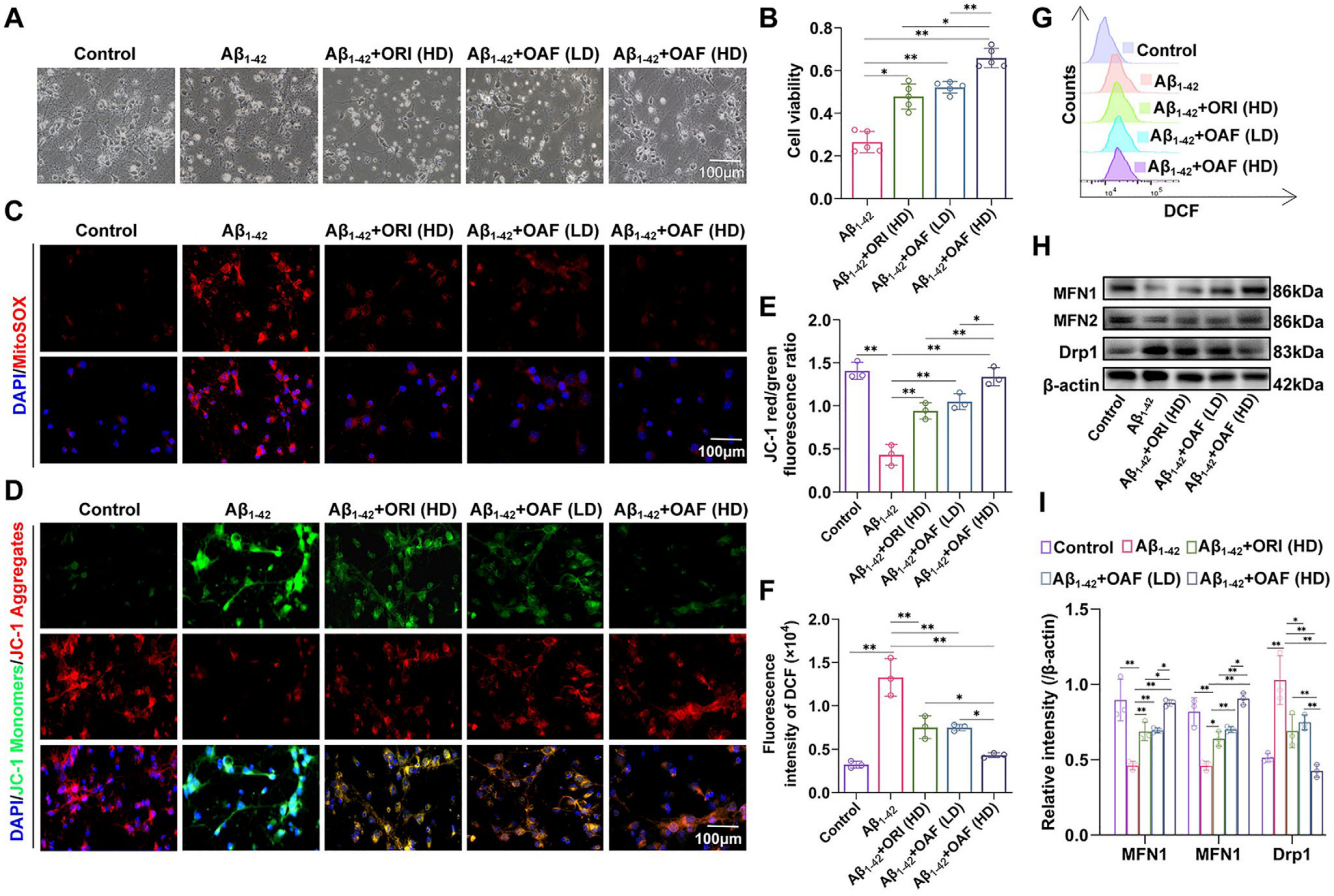
OAF alleviated mitochondrial damage and apoptosis of primary neurons in the liver–brain inflammatory microenvironment. (A) The effect of OAF on the morphology of primary neurons in liver–brain microenvironment model. (B) The effect of OAF on the cell viability of Aβ_1–42_‐injured mice primary neurons (*n* = 5). (C) LSCM images of the effect of ORI, OAF on the intracellular mitochondrial superoxide level in primary neurons in liver–brain microenvironment model. (D,E) LSCM images and analysis of the effect of ORI, OAF on the intracellular mitochondrial membrane potential in primary neurons in liver–brain microenvironment model (*n* = 3). (F,G) Flow cytometry analysis of the effect of ORI, OAF on the intracellular ROS level in primary neurons in liver–brain microenvironment model (*n* = 3). (H) Western blot analysis of the effect of ORI, OAF on mitochondrial dynamics‐related proteins in primary neurons in liver–brain microenvironment model. (I) Semiquantitative analysis of mitochondrial dynamics‐related proteins in panel H (*n* = 3). HD: 10 µm OAF (molar equivalent of ORI). LD: 5 µM OAF (molar equivalent of ORI). Data were presented as mean ± SD. ^*^p < 0.05, ^**^p < 0.01. Statistical significance was calculated via one‐way ANOVA with Tukey's test.

Evidence suggests that the progression of AD is ultimately driven by neuronal loss, which is jointly mediated by neuroinflammation and neuronal apoptosis [47]. In liver–brain microenvironment model, treatment with Aβ_1_–_42_ induced pronounced somatic atrophy, blurred cellular boundaries, and shortened and fragmented axonal and dendritic structures in primary neurons within the recipient chamber. Following OAF intervention, dendritic length exhibited concentration‐dependent restoration alongside improved cellular morphology, suggesting a facilitative role of OAF in neuronal structural repair in liver–brain microenvironment model (Figure 4A). Moreover, Aβ_1_–_42_ exposure reduced neuronal viability, whereas OAF treatment significantly enhanced neuron survival rates (Figure 4B), indicating that OAF effectively counteracted neuronal apoptosis induced by Aβ_1_–_42_ accumulation.

As highly metabolically active cells, neurons rely heavily on mitochondrial energy supply for critical functions such as action potential propagation and synaptic transmission. Thus, the structural and functional integrity of mitochondria is essential for inhibiting neuronal apoptosis [48, 49]. In liver–brain microenvironment model, Aβ_1_–_42_ significantly reduced mitochondrial membrane potential and markedly elevated levels of ROS and mitochondrial superoxide anions in primary neurons, suggesting that Aβ_1_–_42_ promoted neuronal apoptosis by inducing mitochondrial damage and oxidative stress. Treatment with OAF restored mitochondrial membrane potential and significantly decreased the level of ROS and superoxide anion, indicating that OAF exerted neuroprotective effects by alleviating mitochondrial dysfunction and oxidative stress (Figure 4C–G). Mitochondrial dynamics plays a crucial role in maintaining mitochondrial morphology and function, The fusion of mitochondria facilitates functional complementation and stress response, while fission of mitochondria promotes the clearance of damaged mitochondria [50]. Western blot analysis revealed that Aβ_1_–_42_ increased the expression of Drp1 and decreased that of MFN1/MFN2, indicating excessive mitochondrial fission and impaired fusion. In contrast, OAF treatment specifically downregulated Drp1 and upregulated MFN1/MFN2, thereby reestablishing mitochondrial dynamic balance (Figure 4H,I). Ultimately, OAF significantly increased the OCR of neurons (Figure S18).

Collectively, these results demonstrated that Aβ_1_–_42_ triggered neuronal apoptosis by disrupting mitochondrial dynamics and promoting oxidative stress. OAF counteracted these effects by rectifying the fission/fusion imbalance, inhibiting ROS accumulation, and stabilizing mitochondrial membrane potential, demonstrating the superiority of this delivery system in attenuating neuronal apoptosis and restoring mitochondrial function, thereby offering a promising therapeutic strategy to mitigate AD progression associated with neuronal loss.

### 2.5 OAF Simultaneously Targets the Brain and Liver via a TfR1‐Mediated Mechanism

**FIGURE 5:**
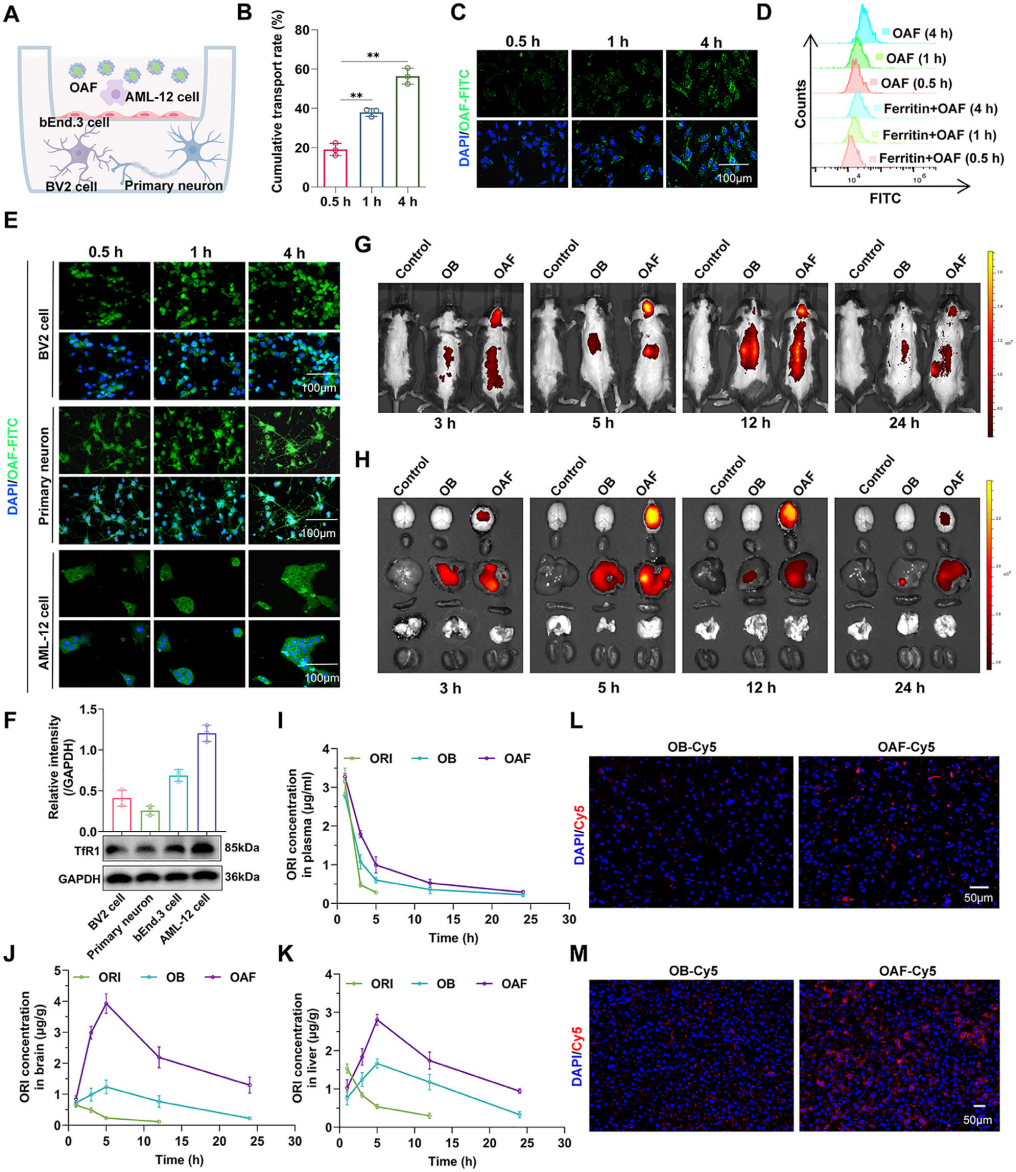
OAF possessed liver–brain targeting capability. (A) Schematic diagram of the uptake of OAF in liver–brain microenvironment. (B) The cumulative transport rate of OAF‐FITC across BBB in liver–brain microenvironment model. (C) LSCM images of the uptake of OAF‐FITC in bEnd.3 cells in liver–brain microenvironment model. (D) Flow cytometry analysis of the uptake of OAF‐FITC in bEnd.3 cells in liver–brain microenvironment model. (E) LSCM images of the uptake of OAF‐FITC in BV2 cells, primary neurons, AML‐12 cells in liver–brain microenvironment model. (F) Western blot analysis of the expression of TfR1 in BV2 cells, primary neurons, bEnd.3 cells, AML‐12 cells in liver–brain microenvironment model. (G) In vivo fluorescence images of the distribution of OB‐Cy5 and OAF‐Cy5 in wild‐type (WT) and 5 × FAD mice after the intravenous injection. (H) Ex vivo fluorescence images of the distribution of OB‐Cy5 and OAF‐Cy5 in main organs of WT and 5 × FAD mice after the intravenous injection. (I) The ORI plasma concentration‐time curve. (J,K) The concentration of ORI in brain and liver tissue in WT and 5 × FAD mice after the intravenous injection. (L) The distribution of OB‐Cy5 and OAF‐Cy5 in cerebral cortex sections. (M) The distribution of OB‐Cy5 and OAF‐Cy5 in liver sections. OB‐Cy5: ORI encapsulated within Cy5‐labeled bovine serum albumin. Data were presented as mean ± SD, *n* = 3. ^*^p < 0.05, ^**^p < 0.01. Statistical significance was calculated via one‐way ANOVA with Tukey's test.

To evaluate the targeting potential of OAF toward the brain and liver, we conducted systematic validation across in vivo and in vitro models. OAF was labeled with fluorescein isothiocyanate (FITC). Treatment with OAF‐FITC for various durations (0, 1, 4 h) did not significantly alter TEER, indicating that it did not disrupt the integrity of the BBB in liver–brain microenvironment model (Figure 5A and Figure S19). Furthermore, fluorescence spectrophotometer analysis revealed that OAF‐FITC effectively crossed the in vitro BBB in a time‐dependent manner (Figure 5B). Flow cytometry and LSCM results demonstrated increased cellular uptake of OAF‐FITC over time in bEnd.3 cells, primary neurons, BV2 cells, and AML‐12 cells in liver–brain microenvironment model (Figure 5C–E). TfR1 is highly expressed in both brain and liver tissues (Figure 5F and Figure S20). When TfR1 was competitively blocked by free ferritin, uptake of OAF‐FITC was significantly reduced across all cell types, confirming that internalization of OAF by key cells within liver–brain microenvironment occurred primarily via TfR1‐mediated endocytosis (Figure 5D and Figure S21).

In vivo imaging in 5 × FAD mice showed that, compared with Cy5‐labeled ORI encapsulated into bovine serum albumin (OB‐Cy5), OAF‐Cy5 exhibited significantly enhanced targeting to both the brain and liver (Figure 5G). Ex vivo organ imaging further confirmed the accumulation of OAF‐Cy5 in the brain and liver, with substantial signals detected at 5 and 12 h after intravenous injection (Figure 5H and Figure S22). Pharmacokinetic analysis indicated that OAF significantly prolonged the circulation half‐life of ORI and increased its concentration in both the liver and brain (Figure 5I–K and Figure S23). LSCM imaging also showed the increased distribution in brain and liver after the treatment of OAF (Figure 5L,M). After entering the brain, OAF exhibited the highest distribution in the cerebral cortex, and was also notably distributed in the hippocampus (Figure S24). Given that Aβ deposition primarily occurs in the cerebral cortex and hippocampus [51, 52], the ability of OAF to accumulate in these regions further supports its potential in effectively clearing Aβ. LSCM imaging revealed significant colocalization of OAF with both TfR1 and the vascular marker CD31 in brain tissue (Figure S25). A similar colocalization pattern of OAF with TfR1 and CD31 was also observed in liver tissue (Figure S26). The above results suggested that OAF could actively target brain and liver tissues via TfR1.

### 2.6 OAF Ameliorated Cognitive Impairment in 5 × FAD Mice

**FIGURE 6:**
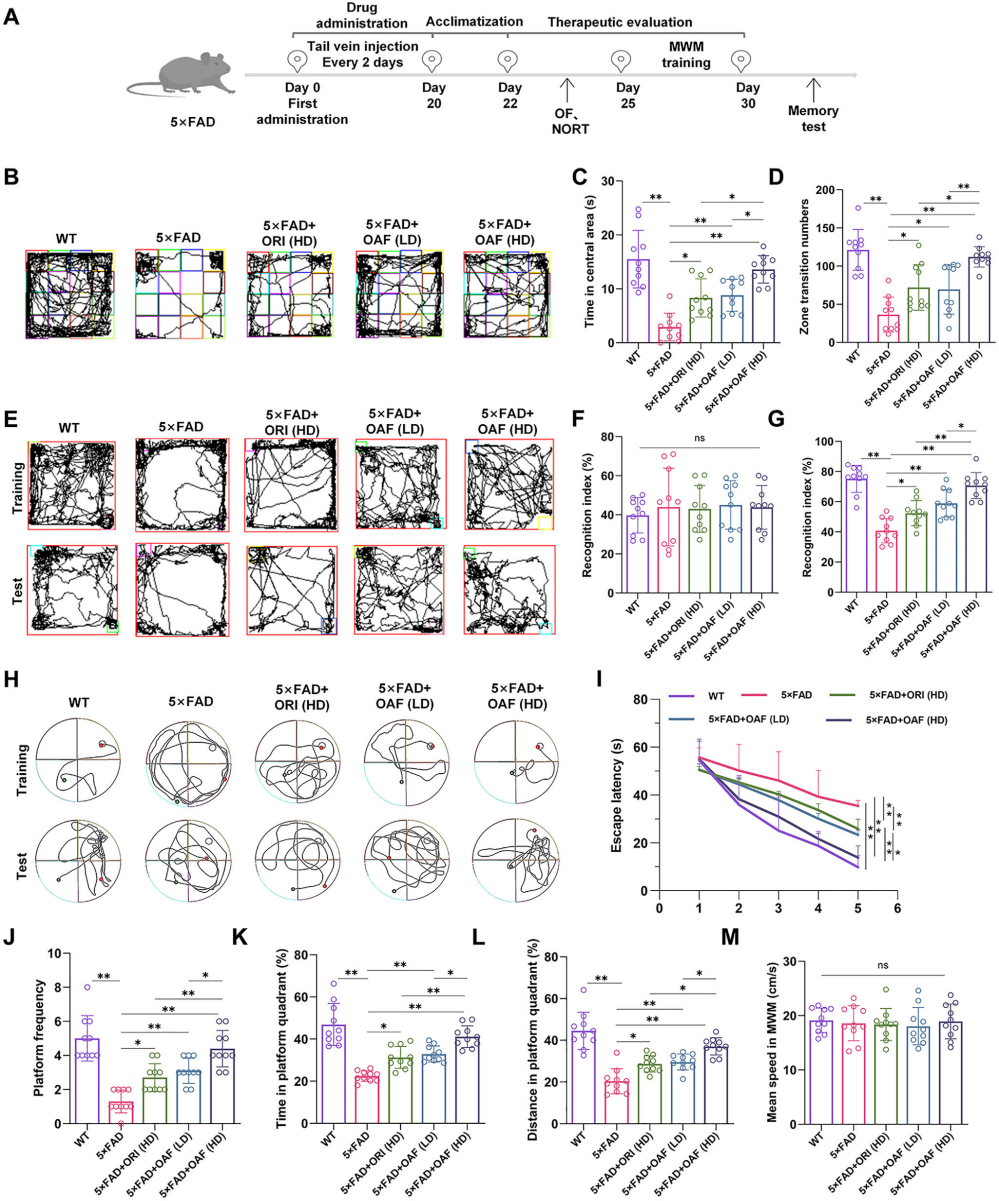
OAF improved cognitive ability of 5 × FAD mice. (A) Time schedule of drug administration and therapeutic evaluation on WT and 5 × FAD mice and their WT littermates. (B) Representative trajectories of mice in OFT experiment. (C) Residence time in central area in OFT experiment. (D) Total numbers of traverses in OFT experiment. (E) Representative trajectories of mice in novel object recognition experiment. (F,G) The cognitive index of mice in NORT experiment. (H) Representative swimming trajectories of mice in MWM experiment. (I) Escape latency within 5 days of training process in MWM experiment. (J–M) The number of crossing platforms, the percentage of time in the target quadrant, the swimming distance and the average swimming speed in the space exploration experiment on the sixth day in MWM experiment. WT: wild‐type. HD: 10 mg/kg (equivalent ORI dose). LD: 5 mg/kg (equivalent ORI dose). Data were presented as mean ± SD, *n* = 10. ns: no significance, ^*^p < 0.05, ^**^p < 0.01. Statistical significance was calculated via one‐way ANOVA with Tukey's test.

Given that this study aims to investigate the targeted modulation of Aβ metabolism by OAF and its improvement on cognitive ability, 5 × FAD mice model was selected primarily because it recapitulates core Aβ pathological features of AD and exhibits rapid, progressive amyloid plaque deposition along with associated cognitive deficits [53, 54]. 5 × FAD mice were administered ORI or OAF via tail vein injection every other day for three weeks. Behavioral and cognitive functions were systematically assessed after the final administration (Figure 6A). Spontaneous locomotor activity was evaluated using the open field test (OFT). No significant differences were observed among groups in total distance moved or average velocity (Figure S27), indicating that general motor function remained unaffected and ruling out potential confounding effects on subsequent behavioral outcomes. Notably, both ORI and OAF treatment significantly increased the time spent and number of entries into the center zone in 5 × FAD mice (Figure 6B‐D), suggesting reduced anxiety‐like behavior and enhanced willingness for independent exploration. Nonspatial memory and recognition were assessed using the novel object recognition test (NORT). OAF treatment dose‐dependently improved the discrimination index in 5 × FAD mice, outperforming free ORI at equivalent doses (Figure 6E–G), indicating superior procognitive recovery effects of OAF.

To further investigate spatial learning and memory, the Morris Water Maze (MWM) test was conducted. During the 5 day navigation training, OAF‐treated 5 × FAD mice showed a significantly faster reduction in escape latency compared to the ORI group. In the probe trial, the OAF group exhibited shorter escape latency, more platform crossings, and longer distance and time spent in the target quadrant (Figure 6H–L). These improvements were dose‐dependent and consistently superior to those in the ORI group, clearly demonstrating that OAF effectively enhanced spatial learning and memory retention in 5 × FAD mice. Furthermore, no significant differences in swimming speed were detected among groups (Figure 6M), confirming that the cognitive improvements were not attributable to the changes in motor function.

These results indicated that OAF treatment effectively alleviated cognitive deficits in 5 × FAD mice, including improvements in spatial learning, memory retention, and cognitive flexibility, with efficacy significantly surpassing that of free ORI. Although these behavioral improvements largely indicated short‐term symptomatic alleviation, they aligned with the concurrent mitigation of key pathological markers, highlighting OAF's promise as a viable intervention strategy for AD‐related cognitive impairment.

### 2.7 OAF Administration Promoted Aβ Clearance in 5 × FAD Mice

**FIGURE 7:**
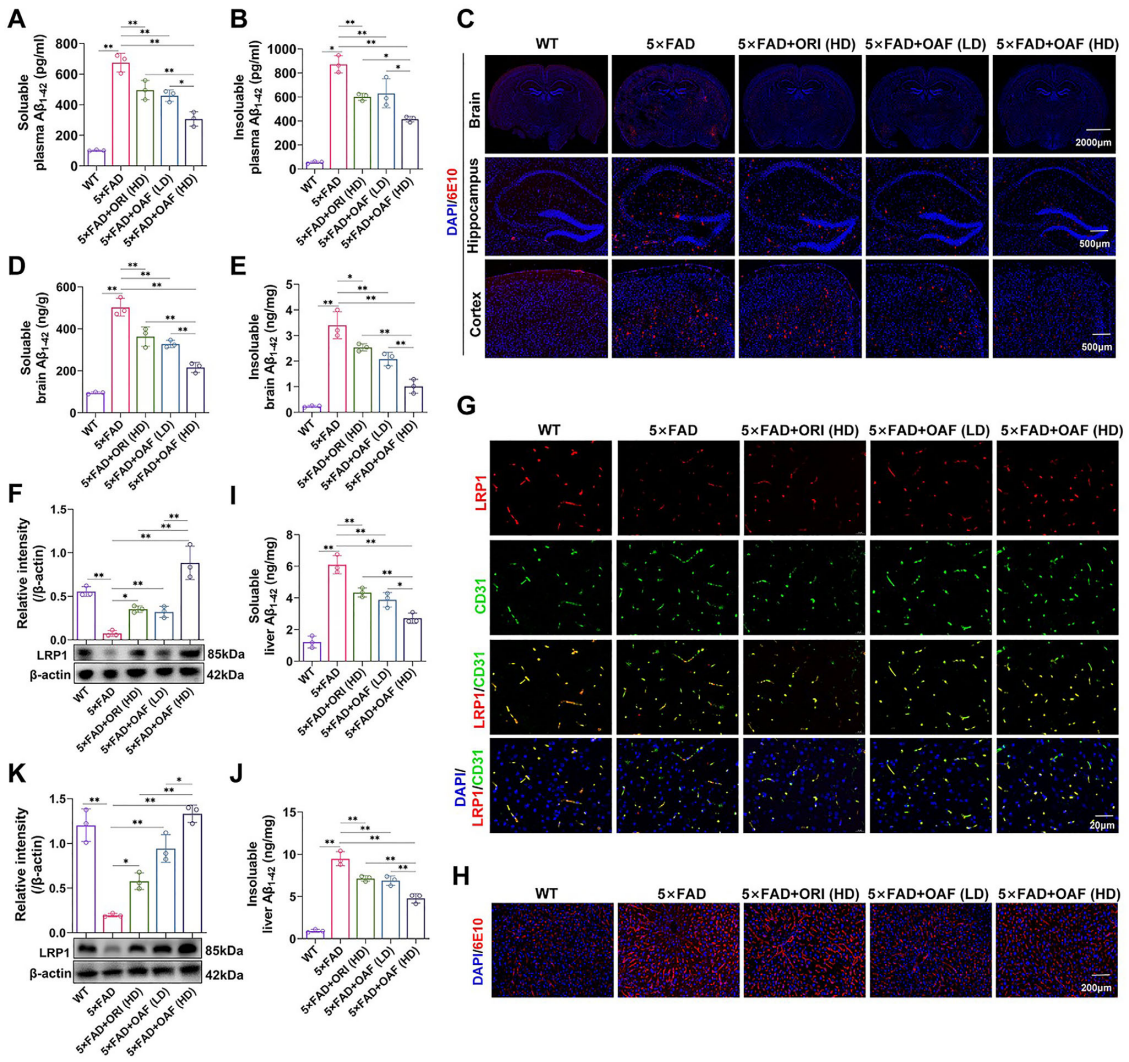
OAF enhanced the capacity of Aβ metabolic in 5 × FAD mice. (A,B) The level of soluble and insoluble Aβ_1−42_ in plasma of WT and 5 × FAD mice. (C) 6E10 staining in hippocampus and cortex tissue of WT and 5 × FAD mice. (D,E) The level of soluble and insoluble Aβ_1−42_ in brain tissues of WT and 5 × FAD mice. (F) Western blot analysis of the expression of LRP1 in brain tissues of WT and 5 × FAD mice. (G) LSCM images of the expression of LRP1 in cortex tissue of WT and 5 × FAD mice. (H) 6E10 staining in liver tissues of WT and 5 × FAD mice. (I,J) The level of soluble and insoluble Aβ_1−42_ in liver tissues of WT and 5 × FAD mice. (K) Western blot analysis of the expression of LRP1 in liver tissue of WT and 5 × FAD mice. WT: wild‐type. HD: 10 mg/kg (equivalent ORI dose). LD: 5 mg/kg (equivalent ORI dose). Data were presented as mean ± SD, *n* = 3. ^*^p < 0.05, ^**^p < 0.01. Statistical significance was calculated via one‐way ANOVA with Tukey's test.

To determine whether the improvement in cognitive function induced by OAF in 5 × FAD mice was associated with Aβ clearance, we first measured the concentration of Aβ in the plasma of 5 × FAD mice using ELISA. The results showed that OAF treatment significantly reduced the levels of both soluble and insoluble Aβ_1_–_42_ in the plasma (Figure 7A,B), suggesting that OAF effectively cleared the accumulated Aβ from the peripheral circulation. Given this peripheral clearance effect, we further evaluated the impact of OAF on cerebral Aβ deposition. Staining with the anti‐Aβ antibody 6E10 showed that OAF treatment significantly reduced both the size and fluorescence intensity of Aβ plaques in the hippocampus and cerebral cortex in 5 × FAD mice (Figure 7C). ELISA further confirmed a significant decrease of soluble and insoluble Aβ_1_–_42_ in brain tissues (Figure 7D,E), indicating that OAF alleviated Aβ pathological burden in the brain.

Subsequent western blot and immunofluorescence analyses demonstrated that OAF markedly upregulated LRP1 expression in cerebrovascular endothelial cells of 5 × FAD mice (Figure 7F,G), confirming that it enhanced LRP1‐mediated transport mechanisms to facilitate Aβ efflux from the brain parenchyma into the bloodstream. Concurrently, immunofluorescence staining of liver tissue indicated markedly decreased Aβ deposition, and ELISA showed significantly reduced Aβ_1_–_42_ levels in liver of OAF‐treated mice (Figure 7H–J), indicating enhanced peripheral Aβ clearance. Importantly, LRP1 expression was also upregulated in the liver (Figure 7K and Figure S28), suggesting improved hepatic uptake and metabolism of Aβ.

These results indicated that downregulation of LRP1 in both the brain and liver contributed to the impaired Aβ clearance and plaque accumulation in 5 × FAD mice. OAF restored Aβ clearance by concurrently upregulating LRP1 expression in these organs, thereby synergistically promoting Aβ efflux from the brain and its metabolic clearance in the liver, ultimately reducing Aβ burden in both the CNS and periphery.

### 2.8 OAF Conferred Neuroprotection by Restoring Mitochondrial Function and Energy Metabolism

**FIGURE 8:**
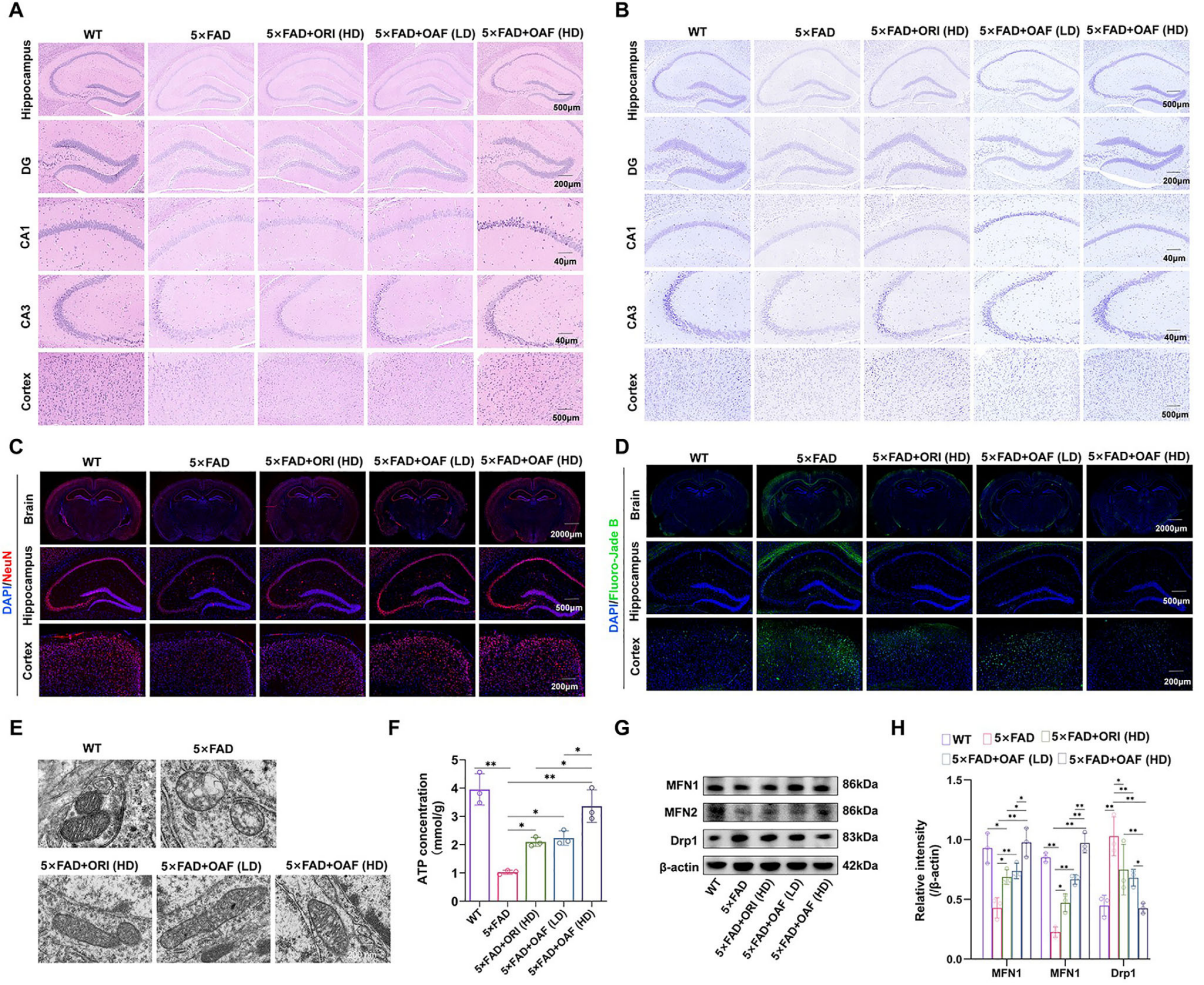
OAF protected neurons in 5 × FAD mice. (A) Hematoxylin and eosin (H&E) staining in mice hippocampus and cortex tissue. (B) Nissl staining in hippocampus and cortex tissue in WT and 5 × FAD mice. (C) NeuN staining in hippocampus and cortex tissue in WT and 5 × FAD mice. (D) FJB staining in hippocampus and cortex tissue in WT and 5 × FAD mice. (E) TEM images of the mitochondria in neuron of hippocampus in WT and 5 × FAD mice. (F) The ATP level in brain tissue in WT and 5 × FAD mice. (G) The expression levels of mitochondrial dynamics‐related proteins in brain tissue in WT and 5 × FAD mice. (H) Semiquantitative analysis of mitochondrial division and fusion‐related proteins in (G). WT: wild‐type. HD: 10 mg/kg (equivalent ORI dose). LD: 5 mg/kg (equivalent ORI dose). Data were presented as mean ± SD, *n* = 3. ^*^p < 0.05, ^**^p < 0.01. Statistical significance was calculated via one‐way ANOVA with Tukey's test.

To investigate the neuroprotective effects of OAF in 5 × FAD mice, we systematically examined the brain histomorphology and neuronal structure. Compared to wild‐type mice, 5 × FAD mice exhibited a significant reduction in neuronal density in the hippocampal CA1/CA3 regions and cerebral cortex. The hippocampal architecture was disorganized with widened extracellular spaces, while cortical neurons showed pyknotic nuclei and necrotic changes. Nissl bodies, granular complexes composed of rough endoplasmic reticulum and ribosomes in the neuronal cytoplasm, serve as key indicators of protein synthesis capacity and metabolic activity, and their status directly reflects neuronal health. In 5 × FAD mice, Nissl bodies were dissolved and significantly reduced, indicating severe neuronal dysfunction. Following OAF treatment, neuronal morphology and arrangement in the hippocampus and cortex were markedly improved, showing more intact structure and clearer cellular boundaries (Figure 8A). The number and integrity of Nissl bodies were notably restored, suggesting recovered biosynthetic and metabolic activity (Figure 8B). Immunostaining for the mature neuronal marker NeuN further confirmed that OAF effectively reduced damage to mature neurons in 5 × FAD mice (Figure 8C). Additionally, staining with Fluoro‐Jade B (FJB), a specific marker for degenerating neurons, revealed that OAF significantly suppressed neurodegenerative changes (Figure 8D).

In terms of energy metabolism, 5 × FAD mice showed a pronounced reduction in ATP levels and exhibited swollen hippocampal mitochondria with disrupted and fragmented cristae, indicating severe bioenergetic deficits. OAF treatment effectively restored mitochondrial ultrastructural integrity (Figure 8E) and significantly increased ATP levels in both the hippocampus and cortex (Figure 8F), demonstrating functional recovery of mitochondrial energy production. Western blot analysis revealed decreased the expression of mitochondrial fusion proteins MFN1 and MFN2, along with the increased expression of fission protein Drp1 in the hippocampus of 5 × FAD mice, suggesting impaired mitochondrial dynamics and enhanced fragmentation. OAF treatment successfully rebalanced mitochondrial dynamics by upregulating expression of MFN1/MFN2 and downregulating expression of Drp1 (Figure 8G,H).

These results demonstrated that OAF exerted neuroprotective effects by restoring mitochondrial ultrastructure, improving energy metabolism, rebalancing mitochondrial fusion‐fission dynamics, enhancing neuronal structural integrity, and ultimately inhibiting apoptosis in affected brain regions of 5 × FAD mice.

### 2.9 OAF Modulated NLRP3 Inflammasome‐Mediated Inflammation Through Brain–Liver Dual Targeting

**FIGURE 9:**
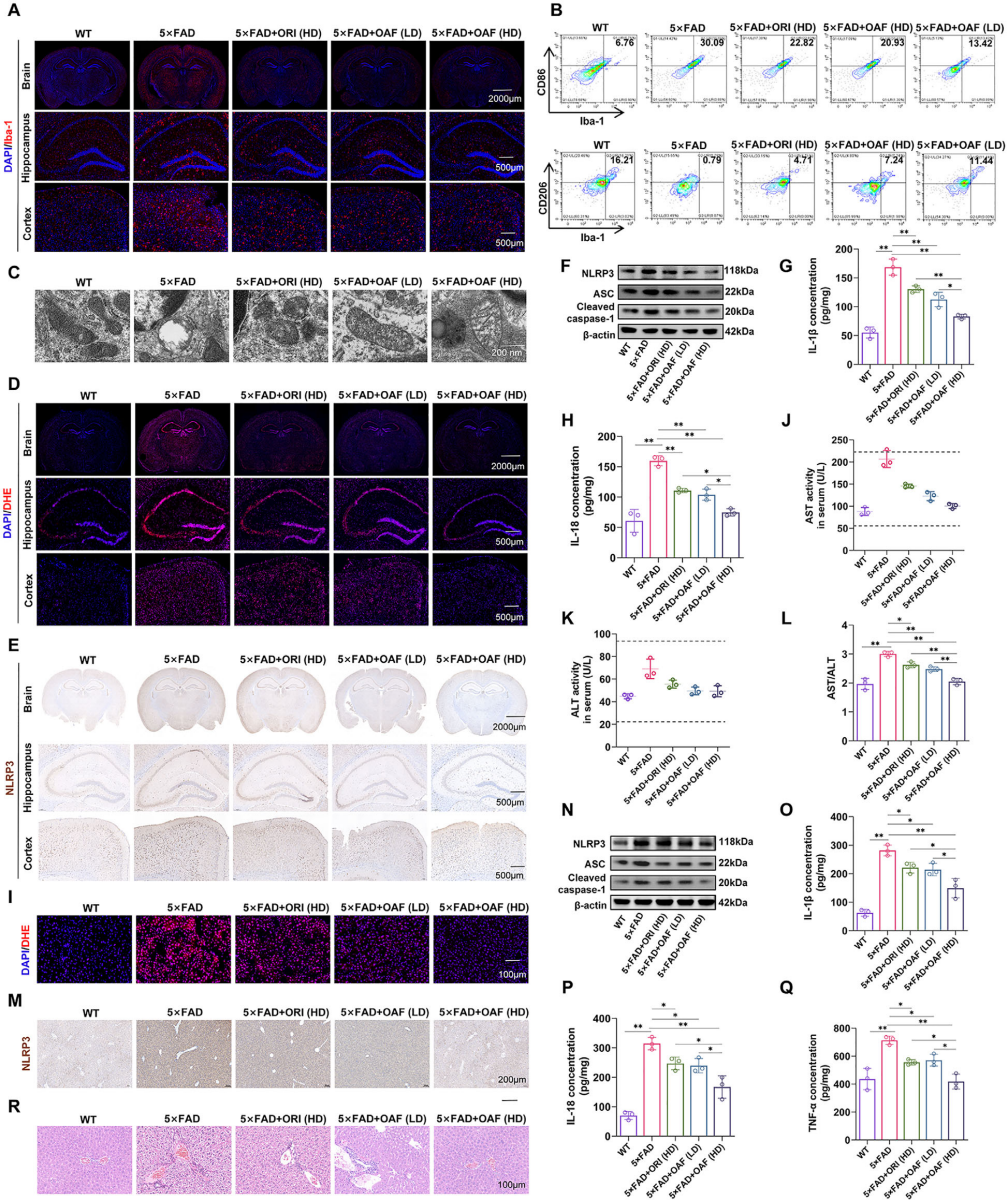
OAF ameliorated inflammatory and oxidative stress in the liver and brain of 5 × FAD mice. (A) Iba‐1 staining in the hippocampus and cortex tissue of WT and 5 × FAD mice. (B) Flow cytometry analysis of M1 and M2 microglia in brain tissues of WT and 5 × FAD mice. (C) TEM images of the mitochondria in hippocampus microglia of WT and 5 × FAD mice. (D) DHE staining in the hippocampus and cortex tissue of WT and 5 × FAD mice. (E) Immunohistochemistry analysis of the expression of NLRP3 in the hippocampus and cortex tissue of WT and 5 × FAD mice. (F) Western blot analysis of the expression of NLRP3 inflammasome‐related proteins in brain tissues of WT and 5 × FAD mice. (G) IL‐1β levels in brain tissues of WT and 5 × FAD mice. (H) IL‐18 levels in brain tissues of WT and 5 × FAD mice. (I) DHE staining in the liver tissues of WT and 5 × FAD mice. (J–L) The hepatorenal functions of WT and 5 × FAD mice. (M) Immunohistochemistry analysis of the expression of NLRP3 in the liver tissues of WT and 5 × FAD mice. (N) Western blot analysis of the expression of NLRP3 inflammasome‐related proteins in liver tissues of WT and 5 × FAD mice. (O) IL‐1β levels in liver tissues of WT and 5 × FAD mice. (P) IL‐18 levels in liver tissues of WT and 5 × FAD mice. (Q) TNF‐α levels in liver tissues of WT and 5 × FAD mice. (R) H&E staining in mice liver tissue. WT: wild‐type. HD: 10 mg/kg (equivalent ORI dose). LD: 5 mg/kg (equivalent ORI dose). Data were presented as mean ± SD, *n* = 3. ^*^p < 0.05, ^**^p < 0.01. Statistical significance was calculated via one‐way ANOVA with Tukey's test.

Consistent with our previous findings in vitro, we demonstrated that cerebral Aβ deposition activated microglia and promoted their polarization toward the proinflammatory M1 phenotype in 5 × FAD mice. This led to the release of large quantities of inflammatory cytokines and suppression of the reparative M2 phenotype, ultimately disrupting immune homeostasis in the brain. OAF treatment significantly reduced the proportion of M1‐microglia while increasing that of M2‐microglia in the brains of 5 × FAD mice (Figure 9A,B and Figure S29), indicating its effective modulation of the neuroimmune microenvironment. Further dual immunofluorescence staining confirmed that OAF treatment markedly reduced NLRP3 expression within microglia (Figure S30). TEM further demonstrated that OAF administration alleviated mitochondrial swelling and restored cristae structure in microglia (Figure 9C). Dihydroethidium (DHE) staining showed that OAF markedly reduced ROS levels in the hippocampal and cortical regions (Figure 9D). Additionally, immunohistochemistry and western blot analysis consistently indicated that OAF significantly suppressed the expression of NLRP3, ASC, and cleaved caspase‐1, and reduced levels of the downstream inflammatory cytokine IL‐1β, IL‐18, and TNF‐α (Figure 9E–H and Figure S31). Together, these results demonstrated that OAF markedly attenuated neuroinflammation by mitigating mitochondrial damage and oxidative stress.

In the liver, DHE staining showed that OAF also significantly decreased ROS levels in 5 × FAD mice (Figure 9I). 5 × FAD mice exhibited abnormal activities of alanine aminotransferase (ALT) and aspartate aminotransferase (AST), as well as an imbalanced ALT/AST ratio, suggesting impaired liver function. OAF treatment restored these parameters to near‐normal physiological levels (Figure 9J–L), indicating a systemic improvement in metabolic homeostasis. Western blot and immunohistochemical analyses revealed that OAF significantly inhibited the activation of the NLRP3 inflammasome in the liver (Figure 9M,N and Figure S32). Correspondingly, the IL‐1β, IL‐18, and TNF‐α levels in serum and liver were significantly decreased after OAF treatment (Figure 9O–Q and Figure S33). Hematoxylin and eosin (H&E) staining further confirmed that OAF reduced inflammatory cell infiltration and restored normal hepatocyte morphology (Figure 9R). These findings suggested that OAF not only alleviated local hepatic inflammation and oxidative stress, but also helped to restore Aβ metabolic function in the liver.

### 2.10 OAF Showed Biocompatibility and Long‐Term Application Safety

To evaluate the biosafety of OAF at therapeutically effective doses, we systematically assessed its potential toxic effects. Experimental results showed that serum creatinine (CREA) and blood urea nitrogen (BUN) levels in mice treated with OAF remained within normal physiological ranges, indicating no signs of renal toxicity (Figure S34A,B). Furthermore, evaluation of immune system impact revealed no significant changes in the counts of neutrophils, monocytes, leukocytes, or lymphocytes in peripheral blood (Figure S34C–F), suggesting that OAF did not elicit notable systemic immune reactions at the treatment dose. Histopathological analysis of major organs via H&E staining showed no significant pathological alterations in the heart, spleen, lungs, or kidneys (Figure S35).

Taken together, these in vivo safety evaluations demonstrated that OAF, at therapeutic doses, did not induce significant toxic reactions in vivo, exhibiting favorable biocompatibility and reliable safety profiles. These findings provided an important foundation of OAF for further translational research and potential long‐term application.

## 3 Conclusions

Despite the growing understanding of AD pathogenesis, effective therapeutic interventions remain constrained by limited BBB penetration and inadequate targeting of both cerebral and peripheral pathological processes, particularly the interplay between brain amyloidosis and hepatic metabolic dysfunction. To address this gap, OAF, a brain and liver dual‐targeting nanodelivery system encapsulating the natural compound ORI was formulated. This formulation not only enhanced BBB permeability and optimized pharmacokinetic profiles but also enabled concurrent intervention in both central and peripheral compartments.

Mechanistically, OAF upregulated LRP1 expression in the brain and liver of 5 × FAD mice, thereby promoting Aβ_1__−__42_ efflux from the brain parenchyma to the periphery and facilitating its hepatic clearance, which collectively reduced Aβ deposition in both tissues. Furthermore, OAF attenuated neuroinflammation and systemic inflammation by restoring mitochondrial function and inhibiting NLRP3 inflammasome signaling in the brain and liver. Concurrently, OAF mitigated neuronal apoptosis through modulation of mitochondrial oxidative stress and mitochondrial dynamics. Collectively, these integrated pathways, including enhancing Aβ clearance, resolving inflammation, and counteracting oxidative stress, underpinned the significant improvement in memory and cognitive function observed in 5 × FAD mice, highlighting the therapeutic potential of this multitarget synergistic strategy.

## 4 Limitations and Future Prospects

Despite these promising findings, our study has limitations that merit deeper investigation. First, although the in vitro liver–brain microenvironment model has preliminarily revealed potential interactions between hepatocytes and brain cells under Aβ pathology in a controlled setting, and animal in vivo experiments have further confirmed that OAF acts simultaneously on both the brain and liver by enhancing their respective Aβ clearance functions, the precise bidirectional molecular interplay triggered by this intervention remains incompletely elucidated. This is largely due to the inherent limitation of in vitro systems in recapitulating the 3D tissue architecture, hemodynamic conditions, and systemic immune and metabolic regulatory networks of whole organisms. Future work should therefore employ techniques such as systemic inflammatory mediator monitoring, liver‐derived metabolite analysis, or extracellular vesicle tracking to directly delineate how OAF‐driven liver modulation influences brain pathology, and vice versa. At the mechanistic level, genetic or pharmacological inhibition of LRP1 in relevant cellular and animal models will help to further clarify OAF's exact mechanism of action. Moreover, this study exclusively used the 5 × FAD mice model. Although widely adopted, 5 × FAD mice carry inherent constraints including an abnormally rapid Aβ deposition rate that may not fully reflect the chronic progression of Alzheimer's disease in humans. Thus, additional Alzheimer's disease model systems should be used to confirm the therapeutic effects of OAF and enhance its translational potential. Finally, thorough assessments of long‐term toxicity, immunogenicity, and repeated‐dose safety will be indispensable and critical steps toward advancing this therapeutic strategy into clinical translation.

## 5 Materials and Methods

### 5.1 Materials and Reagents

All chemicals and reagents were obtained from commercial suppliers and used without further purification. ApoFn was purchased from Sigma‐Aldrich (MO, USA). ORI was supplied by Alfabiotech (Chengdu, China). Aβ_1–42_ was purchased from MedChemExpress (Beijing, China). 1,1,1,3,3,3‐Hexafluoro‐2‐propanol (HFIP) and angelicin were obtained from Sigma‐Aldrich (MO, USA). Percoll was sourced from Coolaber (Beijing, China). B27 Plus, HBSS, Neurobasal Plus, and poly‐d‐lysine were acquired from Thermo Fisher Scientific (MA, USA). The anti‐fluorescence quenching mounting medium (with DAPI) and mitochondrial membrane potential detection kit were procured from Servicebio Technology Co., Ltd. (Wuhan, China). RIPA lysis buffer, BCA protein assay kit, SDS‐PAGE sample loading buffer (5×), and ROS assay kit were purchased from Beyotime Biotechnology (Shanghai, China). The mitochondrial superoxide red fluorescent probe, mitochondrial membrane permeability transition pore detection kit, and mitochondrial green fluorescent probe were obtained from Yeasen Biotechnology Co., Ltd. (Shanghai, China). Mouse Aβ_1–42_ ELISA kit, ATP chemiluminescence assay kit, and mouse IL‐1β, IL‐18, and TNF‐α ELISA kit were supplied by Elabscience Biotechnology Co., Ltd. (Wuhan, China). Anti‐6E10 antibody was purchased from BioLegend (CA, USA). Anti‐β actin, anti‐LRP1, anti‐ASC, and anti‐cleaved caspase‐1 antibodies were obtained from Affinity Biosciences (OH, USA). Anti‐Drp1 and anti‐MFN2 antibodies were from Abcam (London, England). Anti‐MFN1 antibody was supplied by Proteintech Group (IL, USA). Anti‐NLRP3 antibody was purchased from Abways (Shanghai, China).

### 5.2 Preparation and Characterization of OAF

OAF nanoparticles were prepared at room temperature. Briefly, 200 µL of ApoFn solution (350 µg/mL) was added to 20 mL of an acetone–water mixture (55:45, v/v) under stirring using a magnetic stirrer for 10 min. Subsequently, 60 mg of ORI was introduced into the ApoFn mixture and stirred for 1 h. The acetone was then evaporated off using an evaporating dish. The resulting solution was filtered through a 0.22 µm membrane filter to remove any insoluble ORI precipitate. The filtrate was transferred to a 5000 Da MWCO ultrafiltration centrifuge tube and centrifuged at 3000 × *g* for 10 min at room temperature. The retained fraction was washed twice with 1 mL of deionized water under the same centrifugation conditions to remove unencapsulated free ORI. The final product retained in the inner chamber of the ultrafiltration device was collected as the OAF nanoparticle suspension. The morphology of OAF was examined by TEM (JEM‐F200, Japan). A sample of OAF suspension was deposited onto a copper grid and air‐dried prior to imaging. Particle size and zeta potential were determined using DLS on a Malvern ZEN 3600 instrument (UK).

### 5.3 Stability Examination

The prepared OAF nanoparticles were divided into two storage groups. One group stored at 25°C and the other group lyophilized and stored at 4°C for over 6 months. The zeta potential, particle size, and PDI of samples from each group were measured by DLS using a Malvern ZEN 3600 instrument. Additionally, OAF was dispersed in 5 % w/v glucose solution or in phosphate‑buffered saline (PBS) containing 10 % v/v FBS. Changes in zeta potential, particle size, and PDI were monitored daily for 7 days.

### 5.4 Biocompatibility Evaluation

OAF solution was serially diluted with normal saline to obtain test solutions at final concentrations of 200, 100, 50, 20, and 2 µm (molar equivalent of ORI, 2.5 mL each). A negative control (2.5 mL normal saline) and a positive control (2.5 mL purified water) were included. Then, 2.5 mL of a 2% v/v red blood cell suspension was combined with an equal volume of each OAF solution or control solution by gentle pipette mixing. The mixtures were transferred into 50 mL centrifuge tubes and incubated statically at 37°C for 3 h. After incubation, samples were centrifuged at 3000 ×  *g* for 5 min. The hemolytic appearance of each group was visually examined and photographed. Subsequently, 1.5 mL of supernatant was aspirated from each tube, and its absorbance was measured at 414 nm using a UV–vis spectrophotometer. All experiments were performed in triplicate.

The hemolysis rate (%) was calculated using the following formula:

$$\mathrm{Hemolysis}\left(\%\right)=\frac{OD_{\mathrm{Experiment}\;\mathrm{group}}-OD_{\mathrm{Negative}\;\mathrm{group}}}{\left(OD_{\mathrm{Positive}\;\mathrm{group}}-OD_{\mathrm{Negative}\;\mathrm{group}}\right)}\times 100\%$$

### 5.5 Drug Loading Content and Release Behavior Assay

Drug loading content was quantified via HPLC (Waters, USA) on an Acquity BEH C_18_ column (1.7 µm, 2.1 × 100 mm) with detection at 239 nm. The mobile phase was methanol‐water (52:48, v/v) at a flow rate of 1 mL/min, with an injection volume of 15 µL.

The in vitro release profile was assessed by loading 2 mL of OAF into a dialysis membrane (MWCO: 1,000 Da), which was then immersed in 10 mL of PBS (pH 7.4, 6.5, or 5.0) and incubated at 37°C with gentle agitation. At predetermined time points over 72 h, 0.1 mL aliquots of the release medium were withdrawn and replaced with an equal volume of fresh prewarmed buffer. The amounts of ORI released was quantified by HPLC.

### 5.6 Cell Culture

BV2 cells were cultured in high‐glucose Dulbecco's modified Eagle medium (DMEM) supplemented with 10% FBS and 1% penicillin–streptomycin. AML‐12 cells were maintained in DMEM/F12 containing 10% FBS, 1% penicillin–streptomycin, ITS supplement (5 µg/mL insulin, 5 µg/mL transferrin, and 5 ng/mL sodium selenite), and 40 ng/mL dexamethasone. bEnd.3 cells were grown in DMEM/F12 supplemented with 10% FBS and 1% penicillin–streptomycin. All cell lines were incubated at 37°C in a humidified atmosphere of 5% CO_2_, with medium changes or passaging performed every 2–3 days.

Primary neurons were isolated from the hippocampi of embryonic day 14–16 C57BL/6 mouse embryos obtained from pregnant specific pathogen‐free (SPF)‐grade mice euthanized under sodium pentobarbital anesthesia (100 mg/kg, i.p.). Cells were seeded onto poly‐d‐lysine‐coated plates and cultured in Neurobasal Plus medium supplemented with 2% B27, 1% L‐glutamine, and 1% penicillin–streptomycin at 37°C with 5% CO_2_. After 24 h, half of the medium was replaced with fresh medium; thereafter, half‐medium changes were performed every other day. Mature neurons were obtained after 7–9 days and used for subsequent experiments.

### 5.7 Preparation of Aβ_1_–_42_ Oligomers

Lyophilized Aβ_1_–_42_ peptide (1 mg) was dissolved in 222 µL of ice‐cold hexafluoro‐2‐propanol (HFIP) in a sealed tube and vortexed thoroughly. The solution was incubated at room temperature for 1 h to obtain 1 mm Aβ_1_–_42_ in HFIP. Aliquots of 27.75 µL were distributed into eight sterile 1.5 mL microcentrifuge tubes. The HFIP was removed under vacuum drying, and the resulting peptide films were stored at −80°C. For oligomerization, one aliquot of peptide film was reconstituted in 5.5 µL dimethyl sulfoxide (DMSO) and sonicated in an ice‐water bath to dissolve completely, yielding a 5 mm Aβ_1_–_42_ solution in DMSO. This solution was diluted with 269.5 µL of ice‐cold sterile PBS, vortexed, and incubated at 4°C for 24 h to form Aβ_1_–_42_ oligomers (100 µm). The oligomer solution was further diluted to desired concentrations in serum‐free culture medium prior to use.

### 5.8 MTT Cytotoxicity Assay

Cells were seeded uniformly into 96‐well plates and incubated for 24 h. The culture medium was then replaced with serum‐free medium containing ORI or OAF. After an additional 24 h of incubation at 37°C in a 5% CO_2_ humidified atmosphere, 20 µL of MTT solution was added into each well, and the plates were incubated for 4 h at 37°C under light‐protected conditions. The medium was carefully aspirated, and 150 µL of DMSO was added to each well to dissolve the formazan crystals. Absorbance (OD value) was measured at 492 nm using a microplate reader (Molecular Devices). Cell viability was calculated relative to untreated controls.

$$\mathrm{Cell}\;\mathrm{viability}\left(\%\right)=\frac{OD_{\mathrm{test}}-OD_{\mathrm{blank}}}{OD_{\mathrm{control}}-OD_{\mathrm{blank}}}\times 100\%$$

### 5.9 Establishment of the Cerebral Microenvironment Model

bEnd.3 cells were seeded into the donor chamber of a Transwell system (1.5 mL, 1 × 10^5^ cells per mL). TEER was monitored using a Millicell ERS volt–ohm meter (Millipore, USA) until values stabilized at approximately 200 Ω·cm^2^, indicating the successful formation of an in vitro BBB. Meanwhile, BV2 cells were plated onto coverslips placed in 24‐well plates (0.5 mL, 1.6 × 10^5^ cells per mL) and cultured until adherence was achieved. The coverslips containing mature primary neurons and BV2 cells were then transferred to the recipient chamber of the established BBB model. The donor and recipient chambers were assembled to form an integrated brain microenvironment model.

### 5.10 Evaluation of Aβ_1_–_42_ Transport in the Cerebral Microenvironment Model

Prepare a neurobasal culture medium containing 2% B‐27 Supplement, without antioxidants, 0.5 mm GlutaMAX‐I, and 1% penicillin–streptomycin as the coculture medium for the cerebral microenvironment, and preheat it to 37°C for use. The culture medium in the recipient chamber of the established brain microenvironment model was replaced with coculture medium containing 2.5 µm Aβ_1_–_42_ or plain coculture medium as control, followed by incubation at 37°C for 24 h. For drug treatment experiments, the donor chamber medium was replaced with DMEM containing either ORI (10 µm), OAF (5 µm), OAF (10 µm), or vehicle control, while recipient chamber received coculture medium with 2.5 µm Aβ_1_–_42_. The system was incubated at 37°C for 24 h. After incubation, the concentrations of Aβ_1_–_42_ in both donor and recipient chambers were quantified using a commercial ELISA kit.

### 5.11 Establishment of the Liver–Brain Microenvironment Model

AML‐12 cells were seeded onto coverslips placed in 24‐well plates at a density of 1.6 × 10^5^ cells per mL (0.5 mL per well) and cultured until full adherence was achieved. The coverslips with AML‐12 cells were then transferred to the donor chamber of the preestablished brain microenvironment model. The donor chamber was assembled with the Transwell recipient chamber to form an integrated liver–brain microenvironment model.

### 5.12 Assessment of Aβ_1_–_42_ Transport in the Liver–Brain Microenvironment Model

Prepare a neurobasal culture medium containing 2% B‐27 supplement, without antioxidants, 0.5 mm GlutaMAX‐I, and 1% penicillin–streptomycin as the coculture medium for the liver–brain microenvironment, and preheat it to 37°C for use. The medium in the recipient chamber of the liver–brain microenvironment model was replaced with coculture medium containing 2.5 µm Aβ_1_–_42_ or control coculture medium, and incubated at 37°C for 24 h. For drug treatment studies, the donor chamber medium was replaced with DMEM containing ORI (10 µm), OAF (5 µm), OAF (10 µm), or vehicle control, while the recipient chamber received coculture medium supplemented with 2.5 µm Aβ_1_–_42_. The model was incubated at 37°C for 24 h. Following incubation, Aβ_1_–_42_ concentrations in both donor and recipient chambers were measured using a commercial ELISA kit.

### 5.13 Flow Cytometry Analysis

Cells were harvested and washed with cold PBS. Following Fc receptor blockade, the cells were incubated with fluorescently conjugated antibodies against surface markers for 30 min at 4°C under light‐protected conditions. For intracellular staining, cells were fixed and permeabilized using a commercial fixation/permeabilization kit prior to antibody incubation. Data were acquired on a flow cytometer (Beckman Coulter Inc, USA) and analyzed using FlowJo software. Unstained and fluorescence‐minus‐one (FMO) controls were included for accurate gating and background subtraction.

### 5.14 Western Blot

Total protein was extracted from cells or tissues using RIPA lysis buffer supplemented with protease and phosphatase inhibitors. The protein concentration was determined with a BCA protein assay kit (Deeyeebio, China). Equal amounts of protein (20–30 µg) were separated by 10% sodium dodecyl sulfate‐polyacrylamide gel electrophoresis (SDS‐PAGE) and transferred onto PVDF membranes (Merck KGaA, Germany). The membranes were blocked with 5% nonfat milk in TBST for 4 h at room temperature and then incubated with primary antibodies at 4°C overnight. After washing with TBST, the membranes were incubated with HRP‐conjugated secondary antibodies for 1 h at room temperature. Protein bands were visualized using an enhanced chemiluminescence (ECL) detection system (Epizyme Biotech, China) and quantified with ImageJ software. β‐Actin was used as an internal control to ensure equal loading.

### 5.15 Immunofluorescence Staining

Cells were fixed with 4% PFA, permeabilized with 0.2% Triton X‐100, and blocked with 5% BSA. After incubation with primary antibodies at 4°C overnight, cells were stained with fluorophore‐conjugated secondary antibodies and DAPI. Coverslips were mounted with antifade reagent, and images were acquired using a LSCM (Nikon A1Si, Japan).

### 5.16 IL‐1β, IL‐18, and TNF‐α Content Measurement

IL‐1β, IL‐18, and TNF‐α content was quantified using a commercial ELISA kit according to the manufacturer's instructions. Each well was loaded with 100 µL of sample or standard, sealed, and incubated at 37°C for 2 h. After washing and tapping dry, 100 µL of detection antibody was added and incubated at 37°C for 1 h. Following another wash, 100 µL of HRP‐conjugated streptavidin was added, and the plate was resealed and incubated at 37°C for 40 min. After a final wash, 100 µL TMB substrate was added and incubated at 37°C for 20 min in the dark. The reaction was stopped with 100 µL stop solution, resulting in a color change from blue to yellow. Absorbance was measured at 450 nm within 5 min after stopping the reaction. Blank‐corrected OD values were averaged from duplicates, and sample concentrations were determined by extrapolation from the standard curve and multiplied by the dilution factor.

### 5.17 Live Cells Detection by Calcein AM Staining

Cells were washed with PBS and incubated with 20 µL of Calcein AM working solution (2 µmol/L) at 37°C for 30 min. Then, 15 µL of CoCl_2_ solution was added to the culture medium, and the cells were further incubated at 37°C for 20 min. After incubation, the cells were collected and resuspended in 0.5 mL of PBS. The fluorescence intensity of Calcein was measured using flow cytometry.

### 5.18 Intracellular ROS Detection

Cells were washed with PBS and incubated with 1 mL of 10 µm DCFH‐DA solution at 37°C for 20 min. After incubation, the cells were collected and resuspended in 0.5 mL of PBS. The fluorescence intensity of DCF was measured by flow cytometry.

### 5.19 Mitochondrial DNA Detection

Cells were washed with PBS and incubated with 2 mL of MitoTracker Deep Red FM working solution (250 nm) at 37°C for 20 min. After staining, the cells were washed again, fixed with 4% paraformaldehyde, and blocked with 5% BSA. Subsequently, the samples were incubated with an anti‐TFAM antibody at 4°C overnight, followed by incubation with a fluorescent secondary antibody for 1 h at room temperature. Finally, the coverslips were mounted using an antifade mounting medium containing DAPI. The colocalization of MitoTracker and TFAM was examined under a.

### 5.20 Mitochondrial Superoxide Levels Assessment

Primary neuronal cells were washed with PBS and incubated with 1 mL of MitoSOX Red mitochondrial superoxide indicator (5 µm) at 37°C for 10 min. Cells were then collected, resuspended in 0.5 mL of PBS, and analyzed by flow cytometry to measure the fluorescence intensity of oxidized MitoSOX‐nucleic acid adducts.

### 5.21 Mitochondrial Membrane Potential Measurement

Primary neuronal cells were washed with PBS and incubated with 1 mL of JC‐1 staining solution at 37°C for 20 min. The culture medium was removed, and cells were washed twice with JC‐1 staining buffer. After additional PBS washes, cells were fixed with 4% paraformaldehyde and blocked with 5% BSA. Coverslips were mounted with an anti‐fade mounting medium containing DAPI. The ratio of red to green fluorescence, indicative of mitochondrial membrane potential, was visualized using a LSCM (Nikon A1Si, Japan).

### 5.22 Oxygen Consumption Rate Measurement

Mitochondrial respiratory function was assessed using the Agilent Technologies Seahorse XF24 Analyzer (USA). BV2 cells were seeded at a density of 4 × 10^5^ cells per well in Seahorse XF24 plates. Primary neurons were plated at 2.5 × 10^4^ cells per well in poly‐d‐lysine‐coated Seahorse XF24 plates. Prior to the assay, cells were equilibrated for 1 h at 37°C in 500 µL of prewarmed Seahorse XF assay medium (pH 7.4) in a non‐CO_2_ incubator. A sensor cartridge, hydrated overnight at 37°C without CO_2_, was loaded into the analyzer. Following three baseline measurements of the OCR, mitochondrial modulators were injected sequentially through the instrument's automated ports. Oligomycin (1.5 µm for BV2 cells, 2 µm for neurons) was injected via Port A to inhibit ATP synthase. FCCP (1 µm for BV2, 0.5 µm for neurons) was then injected via Port B to uncouple oxidative phosphorylation and induce maximal respiration. Finally, a mixture of rotenone (0.5 µm for BV2, 1 µm for neurons) and antimycin A (1 µm for both) was injected via Port C to completely inhibit mitochondrial electron transport. Basal OCR levels of the cells were calculated from the slope of the kinetic curve following the manufacturer's instructions. Data were normalized to cell number.

### 5.23 Animal Model of Alzheimer's Disease

Five‐month‐old male 5 × FAD mice (specific pathogen‐free, SPF) were used as the AD model in this study, with wild‐type (WT) male C57BL/6 mice (SPF) serving as the control group. All animals were supplied by Shanghai Model Organisms Technology Co., Ltd. (Shanghai, China) and housed in the animal facility of Air Force Medical University. All procedures were approved by the Animal Ethics Committee of Air Force Medical University and conducted in accordance with institutional guidelines (IACUC‐20230094). The mice were maintained in a pathogen‐free barrier environment under controlled conditions, which included a 12 h light/dark cycle, constant temperature and humidity, and ad libitum access to food and water.

### 5.24 Pharmacokinetic Assay

C57BL/6 mice were anesthetized with isoflurane, and blood samples were collected from the retro‐orbital venous plexus. Serum was separated, and homogenates of whole brain and liver tissues were prepared. Isopsoralen (IS, 5 µg/mL) was used as an internal standard. Sample pretreatment involved ether extraction, evaporation under nitrogen flow, and reconstitution in methanol. An HPLC method was developed to quantify ORI concentrations in vivo. The chromatographic conditions were as follows: a mobile phase of methanol‐water (46/54, v/v) at a flow rate of 1 mL/min, UV detection at 239 nm, and a run time of 28 min per sample. A standard curve was constructed by plotting the peak area ratio of ORI to IS against known ORI concentrations. 5 × FAD mice were intravenously injected via the tail vein with ORI, ORI@BSA, or OAF. At 1,3,5,12, and 24 h postinjection, serum, brain, liver, heart, spleen, lung, and kidney tissues were collected and homogenized. The distribution of ORI in blood, brain, and liver was determined using the established HPLC method.

### 5.25 In Vivo Biodistribution of OAF

Cy5‐labeled ORI@BSA (OB‐Cy5, ORI encapsulated within Cy5 labeled bovine serum albumin) and OAF (OAF‐Cy5, ORI encapsulated within Cy5 labeled ApoFn) were administered intravenously via the tail vein to 5 × FAD mice. At 3, 5, 12, and 24 h postinjection, the distribution of the fluorescent probes was monitored using an in vivo imaging system (Vieworks, Smart‐LF). After anesthesia with an overdose of sodium pentobarbital (100 mg/kg, i.p.), the mice were euthanized, and the brain, heart, liver, spleen, lungs, and kidneys were collected. Ex vivo fluorescence imaging of the isolated organs was performed using the same imaging system. The brain and liver were fixed in 4% paraformaldehyde and sectioned for further analysis. Immunofluorescence staining was carried out using antibodies against CD31 and TfR1 to label cerebral blood vessels and assess transferrin receptor expression, respectively. Whole‐slide imaging of the fluorescent immunohistochemical sections was conducted using a Pannoramic MIDI II slide scanner (Pannoramic MIDI II, 3DHISTECH).

### 5.26 Animal Treatment Protocol

5 × FAD mice were randomly assigned to four treatment groups and administered via tail vein every other day for a total of 10 injections: ORI (HD, 10 mg/kg), OAF (LD, 5 mg/kg), OAF (HD, 10 mg/kg), or normal saline (vehicle control). WT mice received the same saline vehicle on an identical schedule and served as an additional control group.

### 5.27 Open Field Test

Each mouse was placed in the center of a rectangular open‐field arena (40 cm × 40 cm × 40 cm) with the floor divided into a 4 × 4 grid of squares, and allowed to explore freely for 5 min. The following parameters were recorded: locomotor speed, total distance moved, time spent in the central and peripheral zones, and the total number of squares crossed. After each trial, the arena was thoroughly cleaned with 75% ethanol to eliminate residual odors that might affect subsequent behavioral tests.

### 5.28 Novel Object Recognition Test

Training phase: On the first day, two identical white cubes were placed diagonally in an open‐field arena (40 cm × 40 cm × 40 cm). Each mouse was positioned at the midpoint between the objects and allowed to explore freely for 5 min. The time spent exploring each object was recorded, with exploration defined as the mouse's nose or head being within 1.0 cm of the object. Test phase: On the following day, one white cube was replaced with a novel blue cone. The mouse was again placed at the midpoint between the two objects and allowed to explore for 5 min. The time spent exploring each object was recorded. The recognition index was calculated using the following formula:

$$r\left(\%\right)=\frac{T_{new}}{T_{new}+T_{old}}\times 100\%$$

### 5.29 Morris Water Maze Test

A circular pool measuring 120 cm in diameter and 50 cm in height was filled with water to a depth of 30 cm. The water temperature was maintained at 22 ± 1°C and rendered opaque by the addition of titanium dioxide to ensure a milky appearance. A white platform, 9 cm in diameter, was submerged 1 cm below the water surface to prevent visual detection by the mice. The pool was conceptually divided into four equal quadrants, with the platform consistently placed at the center of the third quadrant. Distinct visual cues of varying shapes and colors were affixed to the walls of each quadrant to aid spatial learning and navigation. Following this setup, both the hidden platform acquisition training and probe trial testing were conducted.

### 5.30 Neuronal Injury Assessment in Mouse Brain Tissues

Coronal sections of the whole brain were subjected to a series of staining procedures to assess neuronal morphology, viability, and damage. H&E staining was performed to evaluate general histoarchitecture and cellular integrity. Nissl staining with cresyl violet was used to identify viable neurons and assess cytoarchitectural abnormalities. For specific neuronal labeling, immunofluorescence staining against NeuN was conducted to visualize mature neurons. Additionally, FJB staining was applied to identify degenerating neurons. After staining, sections were imaged using a whole‐slide scanner, and quantitative analysis of fluorescence intensity was performed using ImageJ software.

### 5.31 Aβ Levels Measurement in Mouse Brain Tissues

Coronal brain sections and liver tissue sections were stained with the 6E10 antibody. Whole‐slide imaging of the immunofluorescent sections was performed using the Pannoramic MIDI II system, and fluorescence intensity was quantified with ImageJ. Brain and liver homogenates were centrifuged at 12000 × *g* for 5 min at 4°C; the supernatant was collected for soluble Aβ_1–42_ measurement, while the pellet was resuspended in formic acid and RIPA buffer, then recentrifuged to obtain the insoluble fraction. For plasma, ethylenediaminetetraacetic acid (EDTA)‑anticoagulated blood was centrifuged at 1500 × *g* for 15 min at 4°C. A 100 µL aliquot of plasma was mixed with an equal volume of precipitating reagent (0.4% sodium phosphotungstate with 20 mm MgCl_2_), incubated at room temperature for 10 min, and centrifuged at 10 000 × *g* for 15 min at 4°C. The precipitate was resuspended with RIPA buffer. Aβ_1–42_ levels in all samples were quantified using Aβ_1–42_ ELISA kit.

### 5.32 Mitochondrial Function Analysis in Mouse Brain Tissue

Following behavioral testing, mice were anesthetized and transcardially perfused with ice‐cold 4% glutaraldehyde via the tail vein. The brain was fixed in situ before careful extraction. The hippocampus was dissected into 1 mm^3^ blocks and immediately immersed in prechilled electron microscopy fixation solution. Ultrathin sections were prepared and examined under a TEM (JEM‐F200, Japan) to assess mitochondrial morphology in neurons and microglia. Hippocampal and cortical tissues were homogenized after euthanasia. Proteins were extracted and quantified using the BCA assay. Western blot was performed to measure the levels of mitochondrial dynamics proteins (MFN1, MFN2, and Drp1). ATP content in the homogenate supernatant was determined using an enhanced ATP assay kit.

### 5.33 Liver Injury Assessment in Mice

Blood samples were collected from the retro‐orbital venous plexus and analyzed using an automated biochemistry analyzer (Chemray 800) to determine the activities of ALT and AST. Following euthanasia, the liver was promptly excised and fixed in 4% paraformaldehyde. Tissue sections were then prepared and subjected to H&E staining for histological evaluation.

### 5.34 In Vivo Safety Evaluation

After drug administration, 5 × FAD mice were anesthetized with isoflurane, and blood samples were collected from the retro‐orbital venous plexus. Half of the blood was placed in heparin‐coated tubes and centrifuged at 3000 × *g* for 15 min at 4°C to obtain plasma. Levels of serum CREA and BUN were measured using an automated biochemistry analyzer. The remaining blood was collected in EDTA‐containing tubes, and immune cell counts in peripheral blood were analyzed using an automated hematology analyzer (Mindray BC‐2800vet). The heart, spleen, lungs, and kidneys were fixed in 4% paraformaldehyde, embedded, and sectioned for H&E staining. Whole‐slide imaging of the stained sections was performed using the Pannoramic MIDI II system for histological evaluation.

### 5.35 Statistical Analysis

Data are presented as the mean ± standard deviation (SD). All statistical analyses and graphing were performed using GraphPad Prism software. For the statistical comparison between the two groups, the Two‐tailed Student's *t* test was performed. For pairwise multiple comparisons, the one‐way analysis of variance (ANOVA) with Tukey's post‐hoc test was used. For comparisons between other groups and a specific control group, the one‐way ANOVA with Dunnett's test was applied. A *p* value of less than 0.05 was considered statistically significant.

All experiments were conducted with a minimum of three biological replicates (*n* ≥ 3), and all measurements were independently repeated at least 3 times to ensure reproducibility.

## Author Contributions

W.S.G., W.T.H., and S.Q. accomplished the all experiments and analyzed the data. Q.F.J. and M.L. assisted with animal experiments. B.L.Z. analyzed the data. D.Z.L., Y.M.W., and S.Y.Z. designed the experiments, wrote, and reviewed the manuscript.

## Conflicts of Interest

The authors declare no conflicts of interest.

## Supporting information




**Supporting File**: advs75154‐sup‐0001‐SuppMat.docx.

## Data Availability

The data that support the findings of this study are available in the supplementary material of this article.
