# Supplementary material for: Brain and Liver Dual‐Targeting Oridonin Nanoparticles to Enhance Aβ Clearance for Alzheimer's Disease Therapy
Source: Adv Sci (Weinh). 2026 Apr 7;13(36):e23458. doi: 10.1002/advs.202523458 (PMC13317628; doi:10.1002/advs.202523458)
Supplement: Supplementary file 1 — Supporting File: advs75154‐sup‐0001‐SuppMat.docx. [file ADVS-13-e23458-s001.docx]

**Supporting Information**


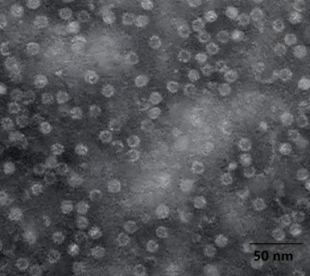


**FIGURE S1** TEM image of ApoFn.


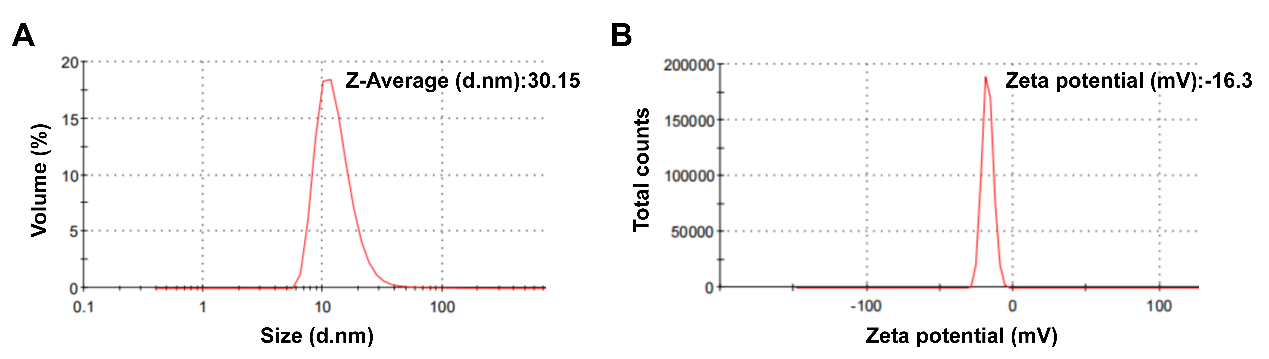


**FIGURE S2** Characterization of ApoFn. (A) Particle size distribution of ApoFn. (B) Zeta potential distribution of ApoFn.

**
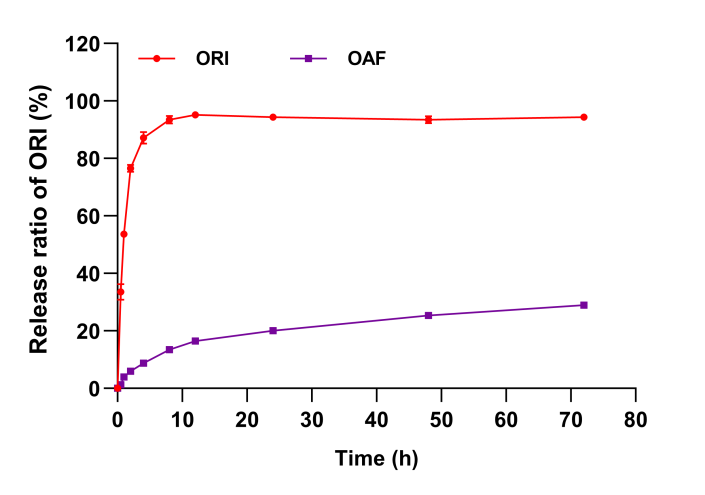
**

**FIGURE S3** Comparison of the *in vitro* release behavior of free ORI and OAF from a dialysis bag in pH 7.4 medium. Data were presented as mean ± SD, n = 3.


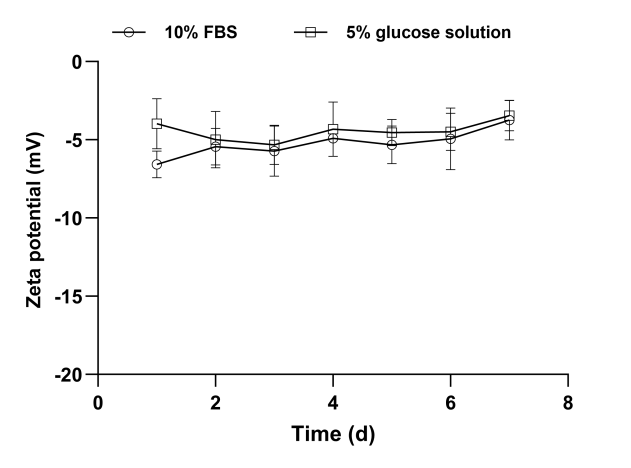


**FIGURE S4** Zeta potentials of OAF in 5% glucose solution or 10% (v/v) FBS solution.


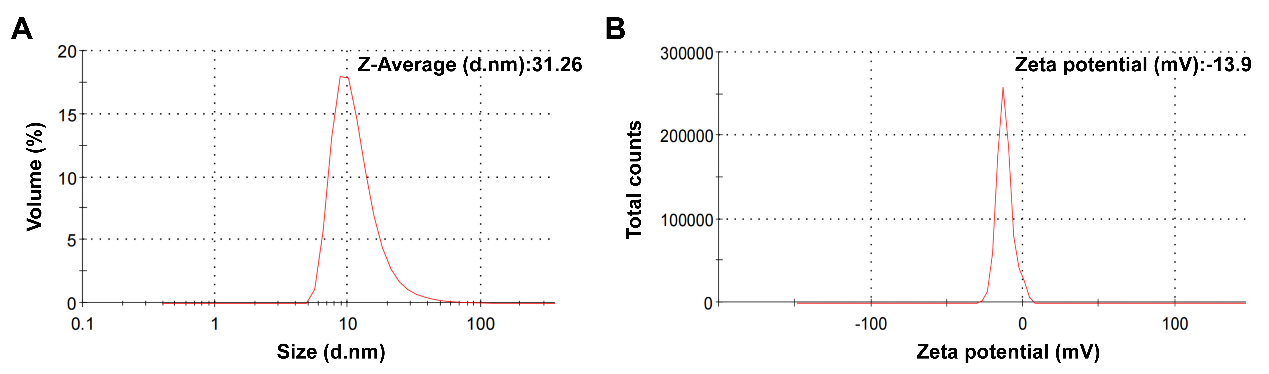


**FIGURE S5** The particle size (A) and zeta potential (B) of OAF after 6 months of storage following lyophilization.


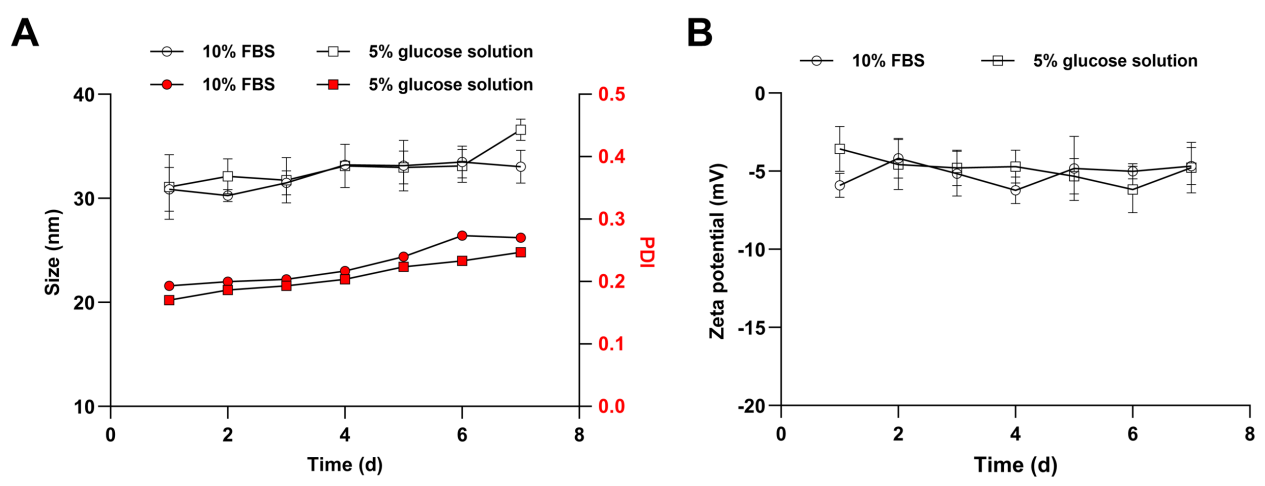


**FIGURE S6** Characteristics of OAF after 6 months of storage following lyophilization. (A) Average particle sizes of OAF in 5% glucose solution or 10% (v/v) FBS solution after 6 months of storage following lyophilization. (B) Zeta potentials of OAF in 5% glucose solution or 10% (v/v) FBS solution after 6 months of storage following lyophilization.


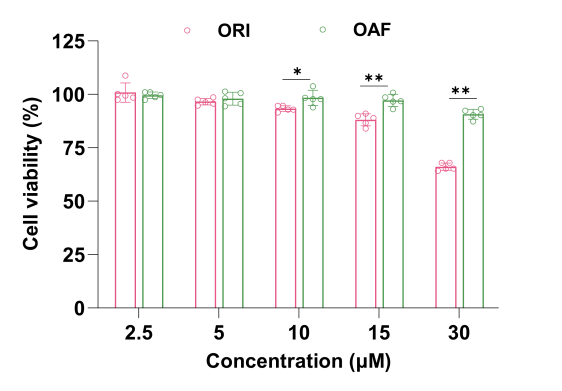


**FIGURE S7** The effect of ORI and OAF on the cell viability of primary neuron. Data were presented as mean ± SD, n = 3. *^*^P* < 0.05, *^**^P*< 0.01. Statistical significance was calculated *via* unpaired two-tailed Student’s t-test.


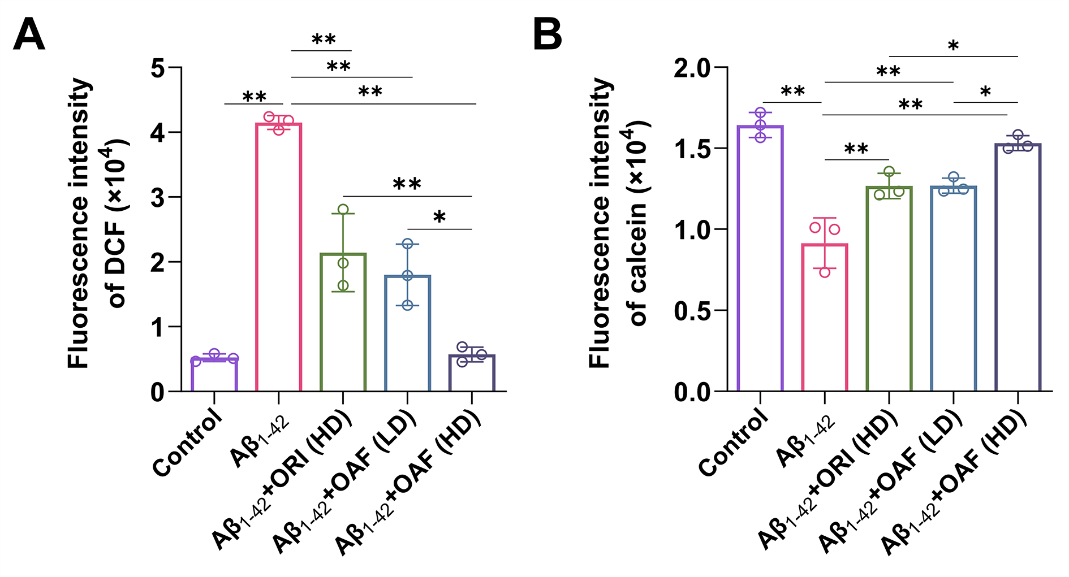


**FIGURE S8** OAF ameliorated the mitochondrial damage of BV2 cells in liver-brain microenvironment model**.** (A) Statistical analysis of the effect of OAF on the intracellular ROS level of BV2 cells in the liver-brain microenvironment model. (B) Statistical analysis of the effect of OAF on the opening of mPTP in BV2 cells in the liver-brain microenvironment model. HD: 10 μM OAF (molar equivalent of ORI). LD: 5 μM OAF (molar equivalent of ORI). Data were presented as mean ± SD, n = 3. *^*^P* < 0.05, *^**^P*< 0.01. Statistical significance was calculated *via* one-way ANOVA with Tukey’s test.


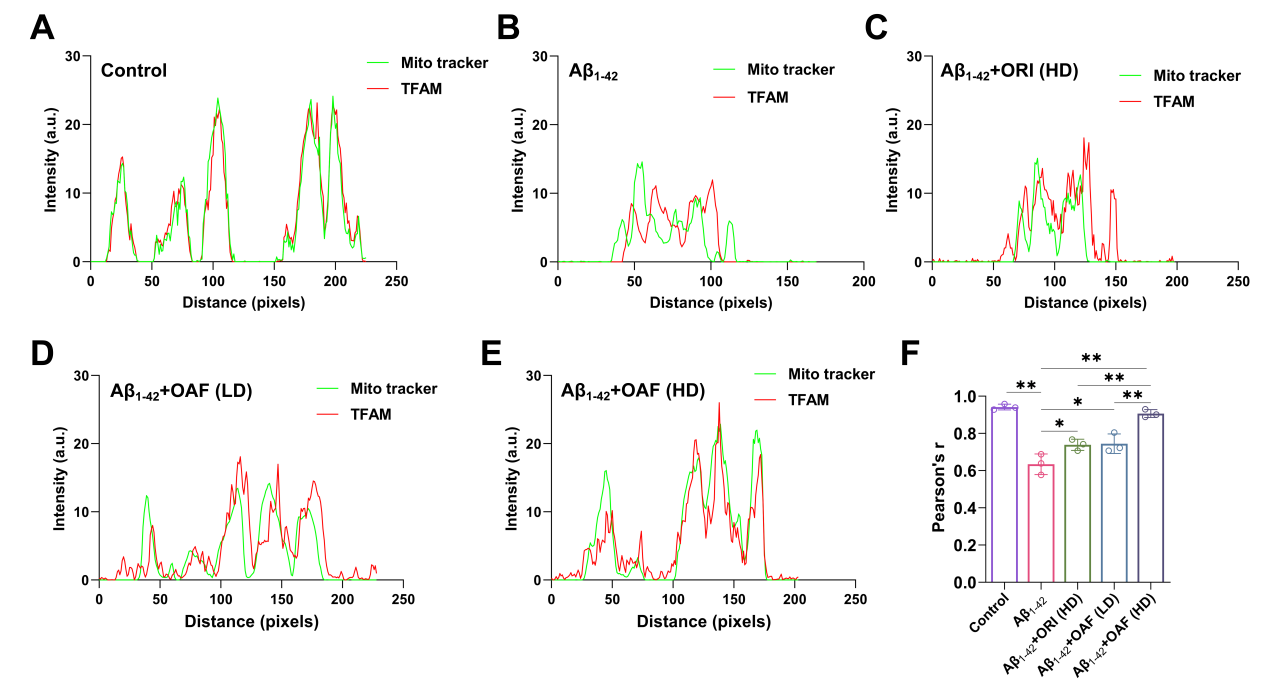


**FIGURE S9** The colocalization analysis of mtDNA and mitochondria in BV2 cells in the liver-brain microenvironment model. (A-E) Colocalization analysis between mtDNA (red) and mitochondria (green) in the control group, Aβ_1-42_ group, Aβ_1-42_ + ORI (HD) group, Aβ_1-42_ + OAF (LD), Aβ_1-42_ + OAF (HD) group. (F) Quantification of colocalization efficiency displayed as Pearson's colocalization coefficient. HD: 10 μM OAF (molar equivalent of ORI). LD: 5 μM OAF (molar equivalent of ORI). Data were presented as mean ± SD, n = 3. *^*^P* < 0.05, *^**^P*< 0.01. Statistical significance was calculated *via* one-way ANOVA with Tukey’s test.


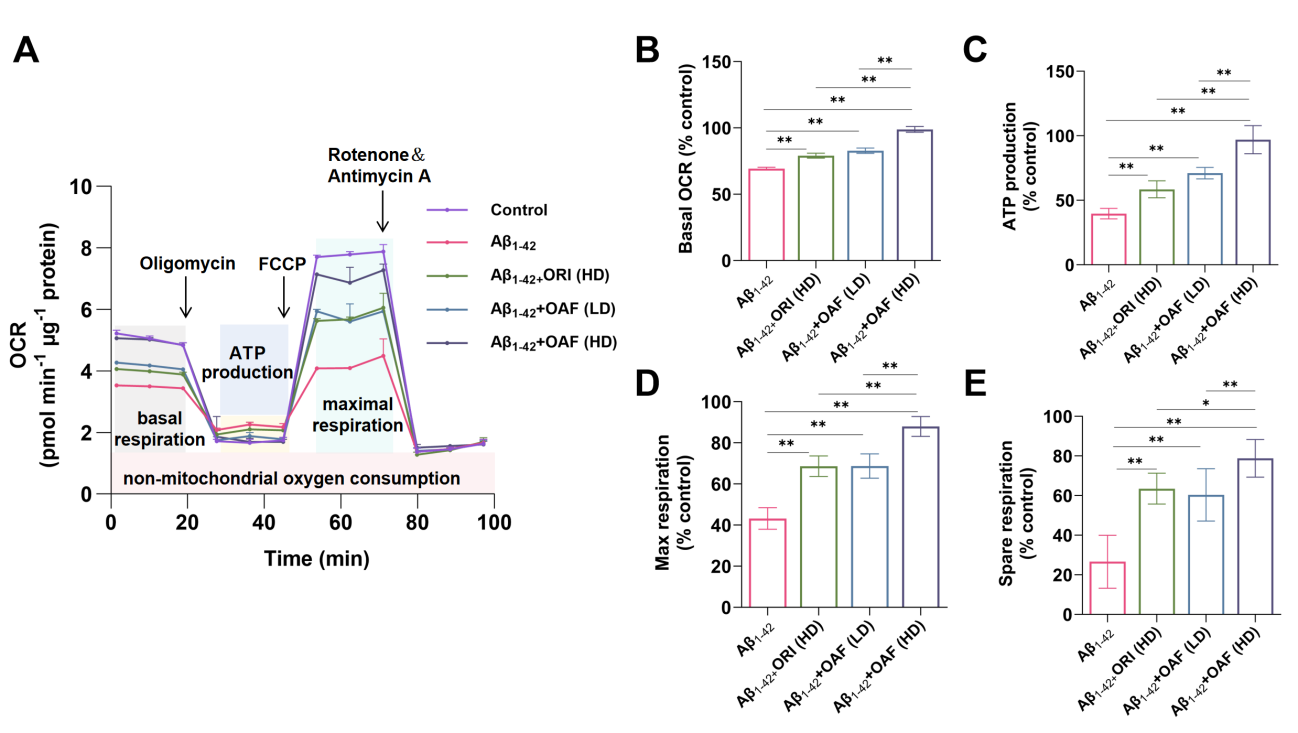


**FIGURE S10** OAF ameliorated mitochondrial respiration of BV2 cells in liver-brain microenvironment model. (A) The OCR measured in BV2 cells in liver-brain microenvironment model. (B) Basal respiration in BV2 cells in liver-brain microenvironment model. (C) ATP production in BV2 cells in liver-brain microenvironment model. (D) Maximal respiration in BV2 cells in liver-brain microenvironment model. (E) Spare respiratory capacity in BV2 cells in liver-brain microenvironment model. HD: 10 μM OAF (molar equivalent of ORI). LD: 5 μM OAF (molar equivalent of ORI). Data were presented as mean ± SD, n = 3. *^*^P* < 0.05, *^**^P*< 0.01. Statistical significance was calculated via one-way ANOVA with Tukey’s test.


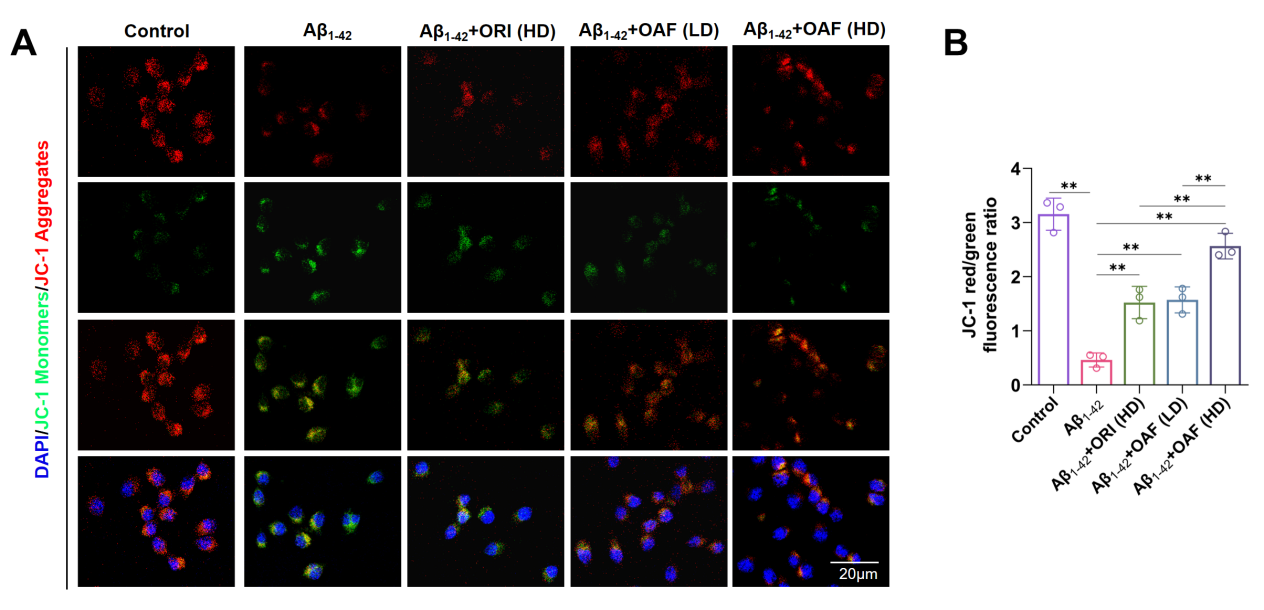


**Figure S11** OAF ameliorated mitochondrial membrane potential of BV2 cells in liver-brain microenvironment model. (A) LSCM images of the effect of ORI, OAF on the mitochondrial membrane potential in BV2 cells in liver-brain microenvironment model. (B) Statistical analysis of panel A. HD: 10 μM OAF (molar equivalent of ORI). LD: 5 μM OAF (molar equivalent of ORI). Data were presented as mean ± SD, n = 3. *^**^P*< 0.01. Statistical significance was calculated via one-way ANOVA with Tukey’s test.


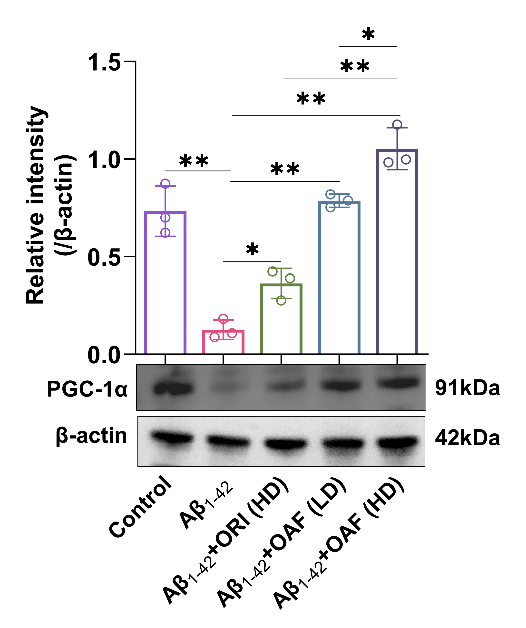


**FIGURE S12** OAF increased the expression of mitochondrial biogenesis protein PGC-1α in BV2 cells in liver-brain microenvironment model. HD: 10 μM OAF (molar equivalent of ORI). LD: 5 μM OAF (molar equivalent of ORI). Data were presented as mean ± SD, n = 3. *^*^P* < 0.05, *^**^P*< 0.01. Statistical significance was calculated via one-way ANOVA with Tukey’s test.


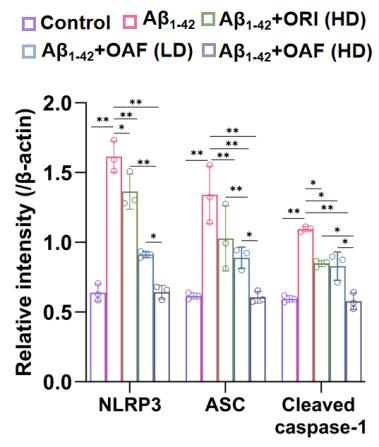


**FIGURE S13** Semi-quantitative analysis of the effect of ORI, OAF on the expression of NLRP3 inflammasome-related proteins in BV2 cells in liver-brain microenvironment model. HD: 10 μM OAF (molar equivalent of ORI). LD: 5 μM OAF (molar equivalent of ORI). Data were presented as mean ± SD, n = 3. ^*^*P* < 0.05, ^**^*P* < 0.01. Statistical significance was calculated *via* one-way ANOVA with Tukey’s test.


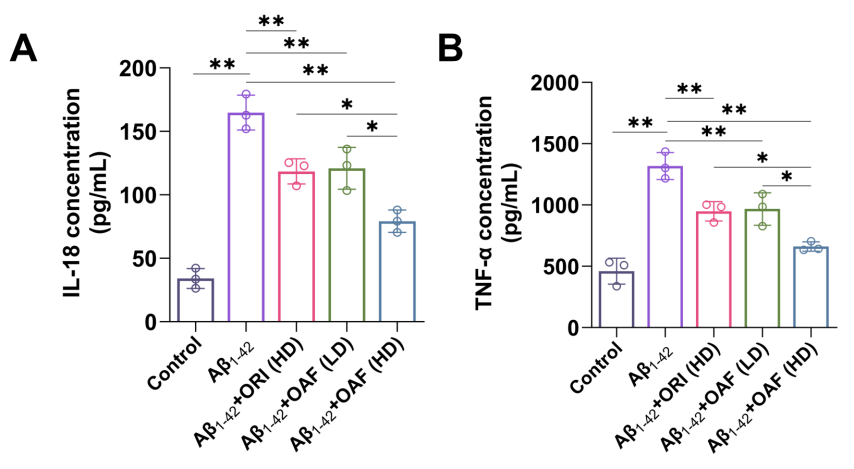


**FIGURE S14** OAF treatment significantly reduced the level of IL-18 (A) and TNF-α (B) in recipient chamber medium in liver-brain microenvironment model. HD:10 μM OAF (equivalent ORI concentration); LD: 5 μM OAF (equivalent ORI concentration). Data were presented as mean ± SD, n = 3. *^*^P <* 0.05, *^**^P* < 0.01. Statistical significance was calculated *via* one-way ANOVA with Tukey’s test.


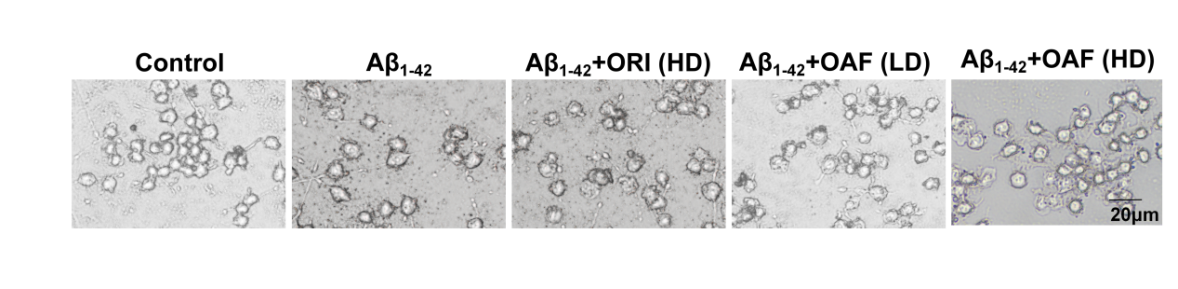


**FIGURE S15** The effect of ORI and OAF on the morphology of BV2 cells in liver-brain microenvironment model. HD: 10 μM OAF (molar equivalent of ORI). LD: 5 μM OAF (molar equivalent of ORI).


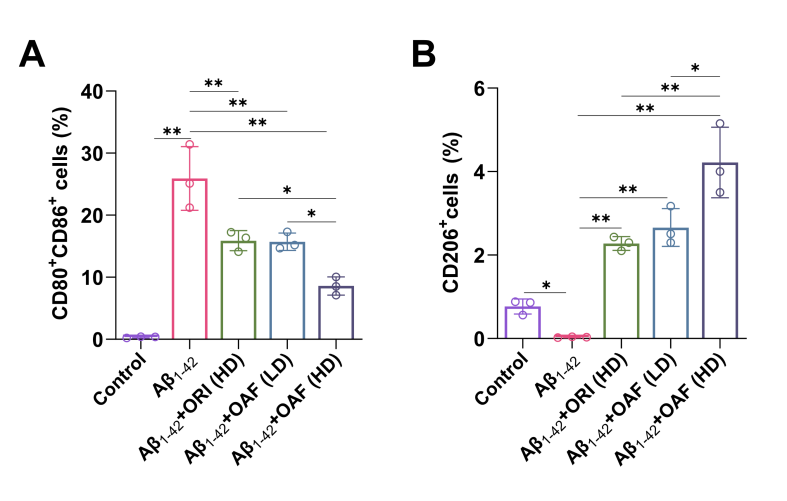


**FIGURE S16** Flow cytometry analysis of M1- and M2-BV2 cells in liver-brain microenvironment model. (A) Statistical analysis on the content of M1-BV2 cells in liver-brain microenvironment model. (B) Statistical analysis on the content of M2-BV2 cells in liver-brain microenvironment model. HD: 10 μM OAF (molar equivalent of ORI). LD: 5 μM OAF (molar equivalent of ORI). Data were presented as mean ± SD, n = 3. *^*^P* < 0.05, *^**^P* < 0.01. Statistical significance was calculated *via* one-way ANOVA with Tukey’s test.


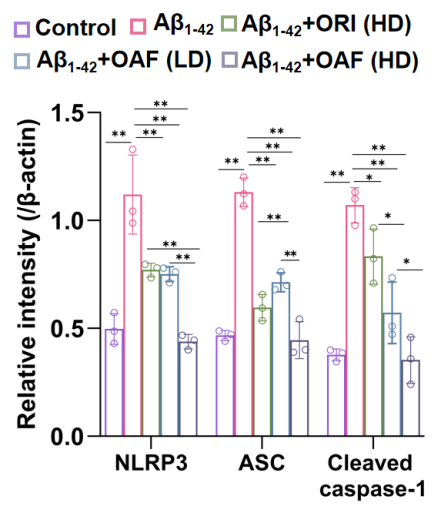


**FIGURE S17** Semi-quantitative analysis of the effect of ORI, OAF on the expression of NLRP3 inflammasome-related proteins in AML-12 cells in liver-brain microenvironment model. HD: 10 μM OAF (molar equivalent of ORI). LD: 5 μM OAF (molar equivalent of ORI). Data were presented as mean ± SD, n = 3. ^*^*P* < 0.05, *^**^P* < 0.01. Statistical significance was calculated *via* one-way ANOVA with Tukey’s test.


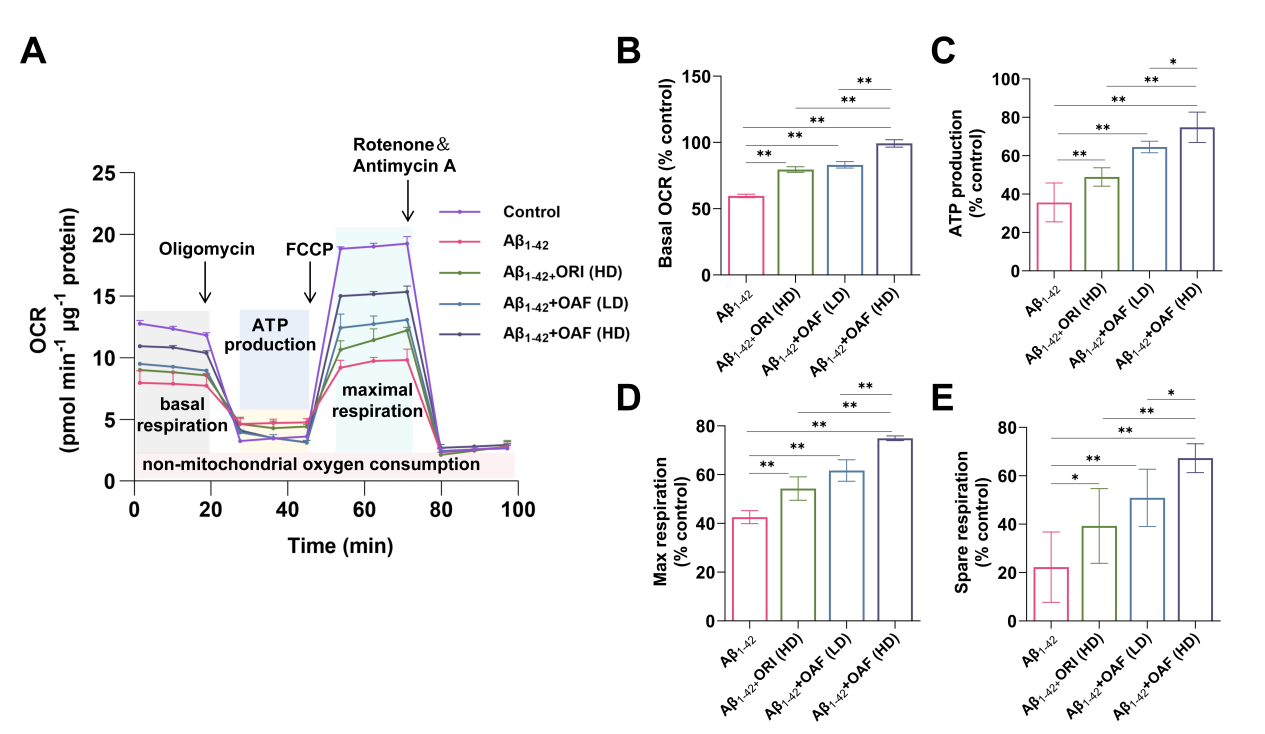


**FIGURE S18** OAF ameliorated mitochondrial respiration in primary neurons in liver-brain microenvironment model. (A) The OCR measured in primary neurons in liver-brain microenvironment model. (B) Basal respiration in primary neurons in liver-brain microenvironment model. (C) ATP production in primary neurons in liver-brain microenvironment model. (D) Maximal respiration in primary neurons in liver-brain microenvironment model. (E) Spare respiratory capacity in primary neurons in liver-brain microenvironment model. HD: 10 μM OAF (molar equivalent of ORI). LD: 5 μM OAF (molar equivalent of ORI). Data were presented as mean ± SD, n = 3. *^*^P* < 0.05, *^**^P* < 0.01. Statistical significance was calculated via one-way ANOVA with Tukey’s test.


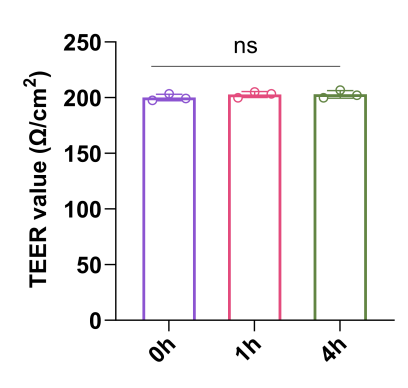


**FIGURE S19** The TEER values between transwell donor chamber and recipient chamber after OAF administration. Data were presented as mean ± SD, n=3. ns: not significant. Statistical significance was calculated *via* one-way ANOVA with Tukey’s test.


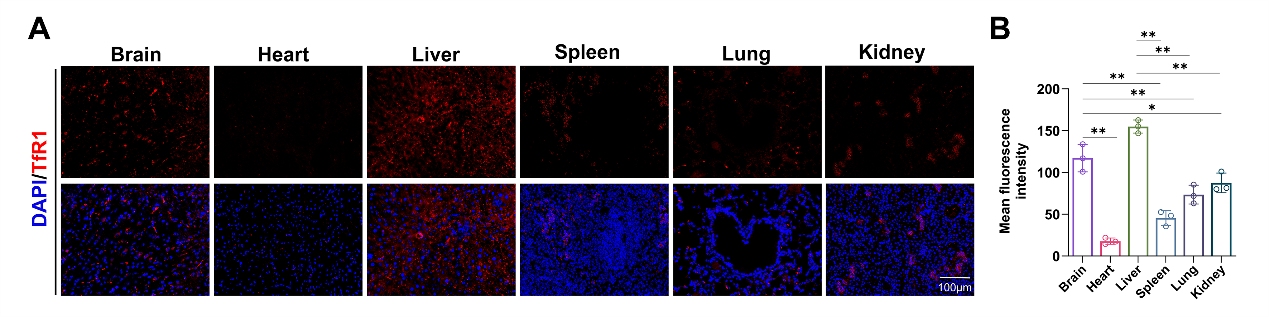


**FIGURE S20** Immunofluorescence detection of TfR1 expression in major organs. (A) LSCM images of TfR1 expression in brain, heart, liver, spleen, lung, and kidney. (B) Statistical analysis of quantitative TfR1 expression results. Data were presented as mean ± SD, n = 3. *^*^P* < 0.05, *^**^P* < 0.01. Statistical significance was calculated *via* one-way ANOVA with Tukey’s test.


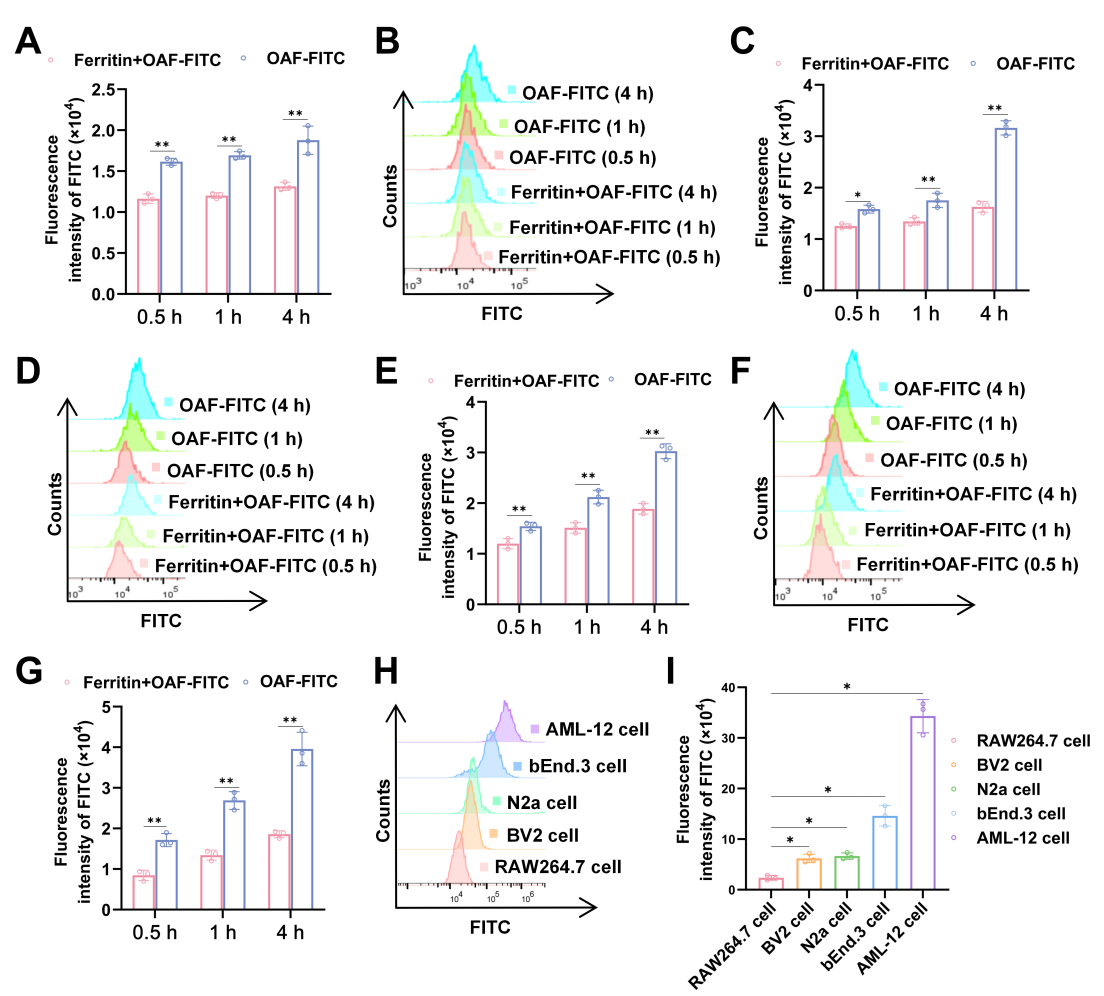


**FIGURE S21** The uptake of OAF by cells in the liver-brain microenvironment. (A) Flow cytometry analysis of the uptake of OAF-FITC in bEnd.3 cells. (B, C) Flow cytometry analysis of the uptake of OAF-FITC in BV2 cells. (D, E) Flow cytometry analysis of the uptake of OAF-FITC in primary neurons. (F, G) Flow cytometry analysis of the uptake of OAF-FITC in AML-12 cells. (H, I) Flow cytometry analysis of the uptake of OAF-FITC in RAW264.7 cells, BV2 cells, N2a cells, bEnd.3 cells, AML-12 cells after 4 h of co-culture. Data were presented as mean ± SD, n=3. *^*^P* < 0.05, *^**^P*< 0.01. Statistical significance was calculated *via* unpaired two-tailed Student’s t-test (A, C and G) or one-way ANOVA with Tukey’s test (I).


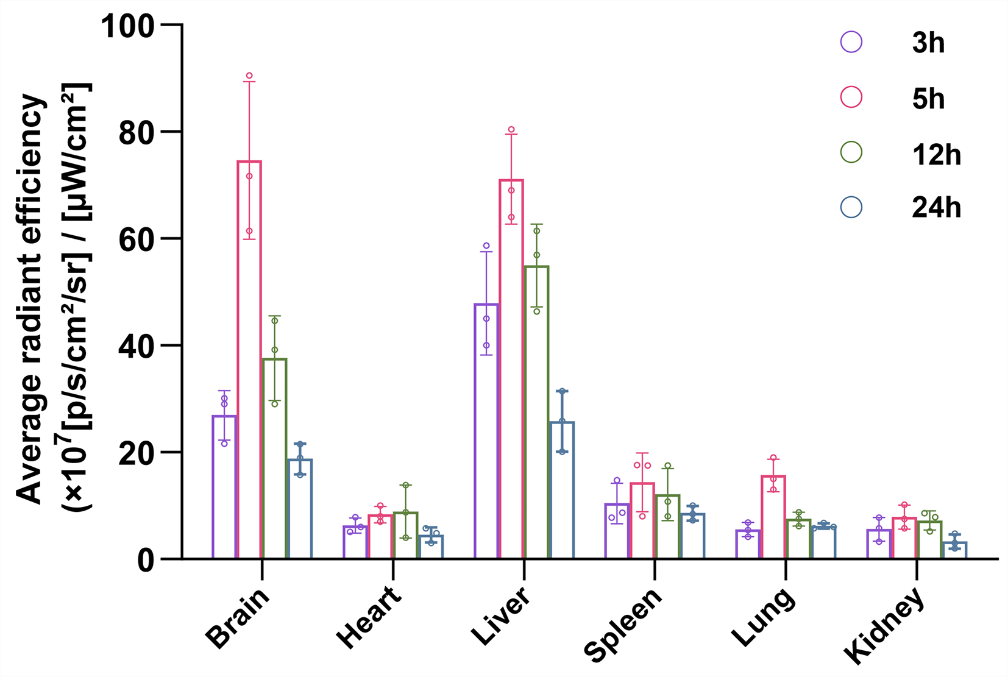


**FIGURE S22** The average radiant efficiency of OAF-Cy5 fluorescence in major organs of 5×FAD mice after the intravenous injection. Data were presented as mean ± SD, n = 3.


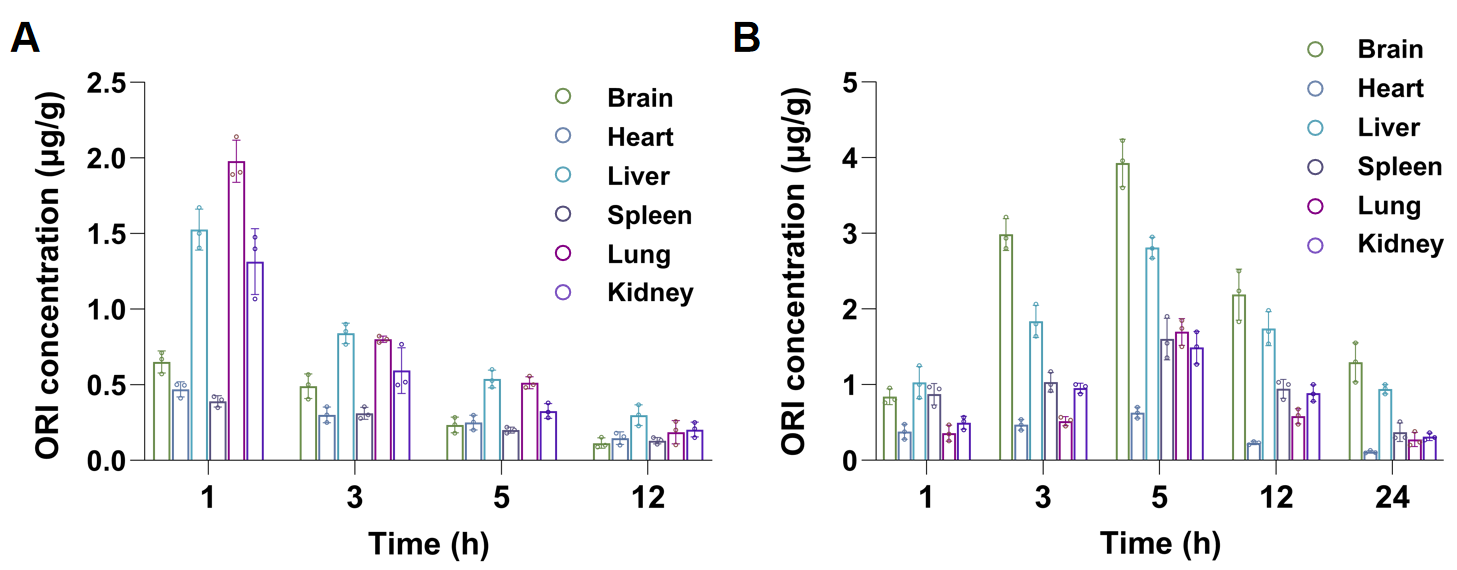


**FIGURE S23** The concentration of ORI in major organs of 5×FAD mice after the intravenous injection. (A) Concentration of ORI in major organs at different time points after the intravenous injection of free ORI. (B) Concentration of ORI in major organs at different time points after the intravenous injection of OAF. Data were presented as mean ± SD, n = 3.


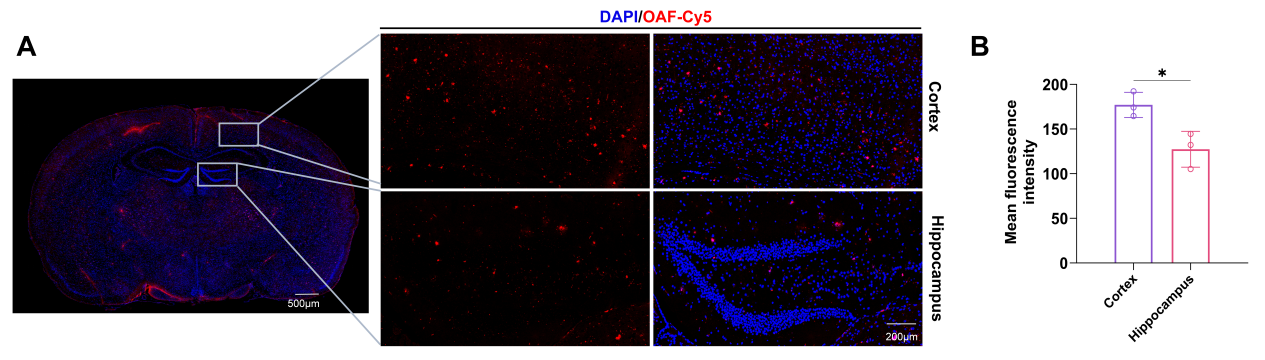


**FIGURE S24** The distribution of OAF-cy5 in the cerebral cortex and hippocampal region. (A) The CLSM image of OAF-cy5 in brain section. (B) Statistical results of fluorescence intensity in panel A. Data were presented as mean ± SD, n = 3. *^*^P* < 0.05, Statistical significance was calculated *via* Two-tailed Student’s t test.


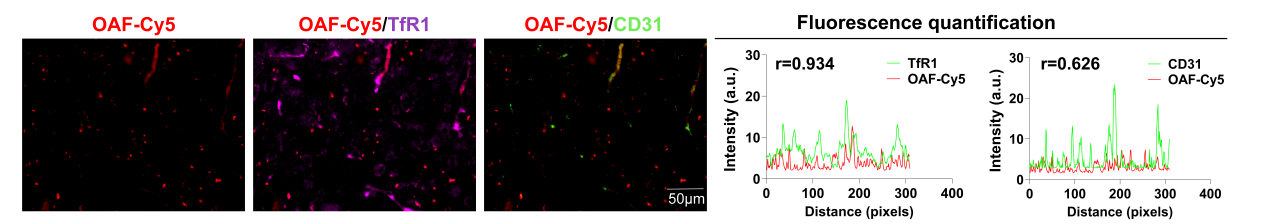


**FIGURE S25** The colocalization analysis between OAF-cy5 (red) and TfR1 (violet) or CD31(green) in the cerebral cortex of 5×FAD mice.


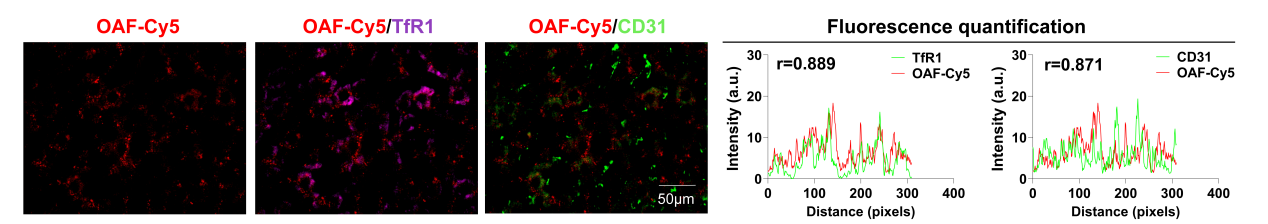


**FIGURE S26** The colocalization analysis between OAF-cy5 (red) and TfR1 (violet) or CD31(green) in the liver of 5×FAD mice.


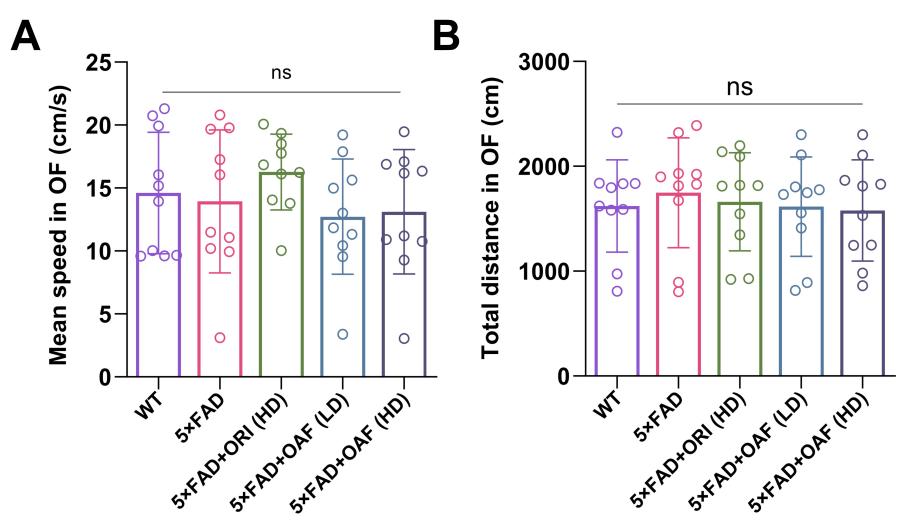


**FIGURE S27** The performance of mice in OFT experiment. (A) Mean speed of mice in OFT experiment. (B) Total moving distance mice in OFT experiment. WT: wild-type. HD: 10 mg/kg (equivalent ORI dose). LD: 5mg/kg (equivalent ORI dose). Data were presented as mean ± SD, n=10, ns: not significant. Statistical significance was calculated *via* one-way ANOVA with Tukey’s test.


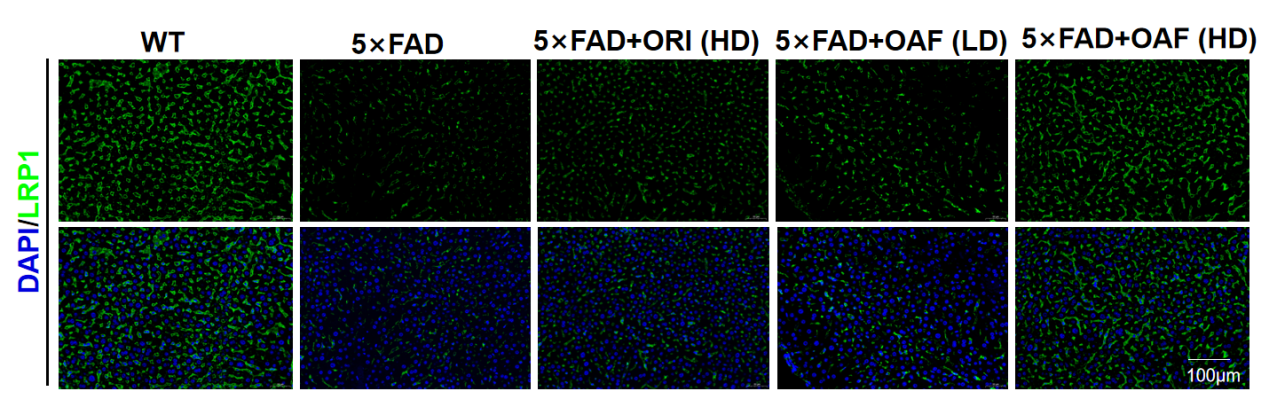


**FIGURE S28** LSCM images of the expression of LRP1 in mice liver. WT: wild-type. HD: 10 mg/kg (equivalent ORI dose). LD: 5mg/kg (equivalent ORI dose).


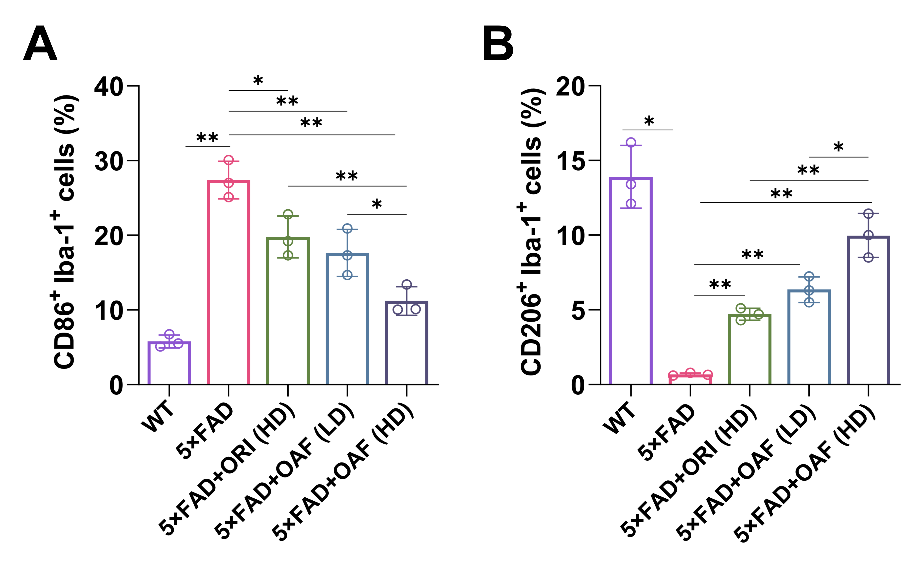


**FIGURE S29** Flow cytometry analysis of M1-and M2-microglia in mice brain tissues. (A) M1-microglia (Iba-1 and CD86 co-positive cells) in mouse brain tissues. (B) M2-microglia (Iba-1 and CD206 co-positive cells) in mouse brain tissues. WT: wild-type. HD: 10 mg/kg (equivalent ORI dose). LD: 5mg/kg (equivalent ORI dose). Data were presented as mean ± SD, n=3. *^*^P* < 0.05, *^**^P*< 0.01. Statistical significance was calculated *via* one-way ANOVA with Tukey’s test.


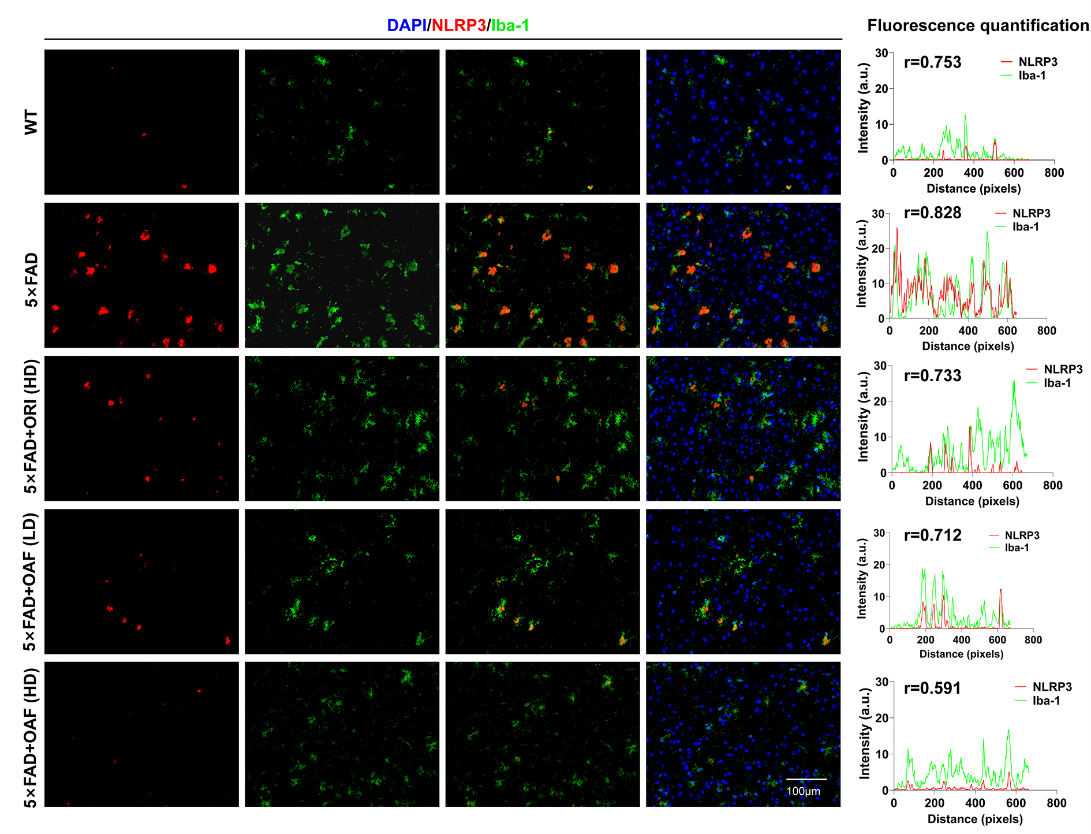


**FIGURE S30** The colocalization analysis between NLRP3 (red) and Iba-1 (green) in mice brain tissues. WT: wild-type. HD: 10 mg/kg (equivalent ORI dose). LD: 5mg/kg (equivalent ORI dose).


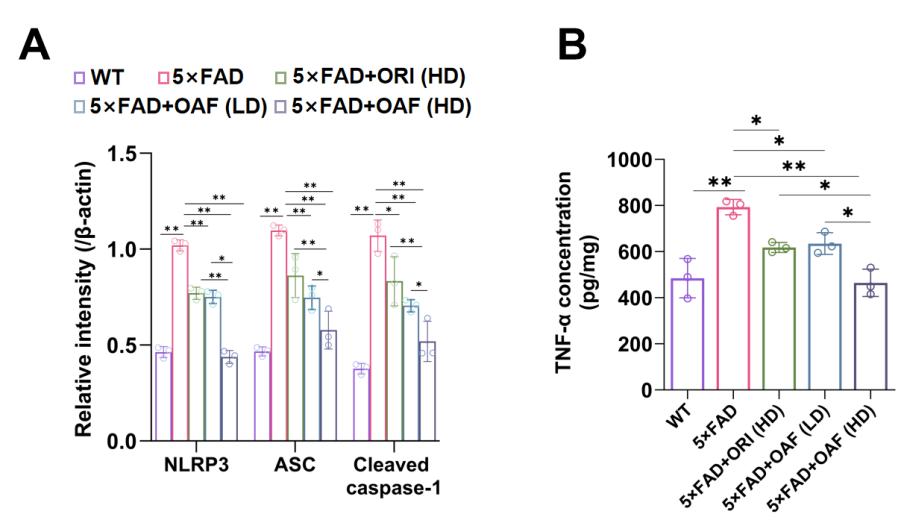


**FIGURE S31** OAF ameliorated the inflammatory in brain tissue of 5×FAD mice. (A) Western blot analysis of the expression of NLRP3 inflammasome-related proteins in mice brain tissues. (B) TNF-α levels in mice brain tissues. WT: wild-type. HD: 10 mg/kg (equivalent ORI dose). LD: 5mg/kg (equivalent ORI dose). Data were presented as mean ± SD, n=3. *^*^P* < 0.05, *^**^P*< 0.01. Statistical significance was calculated *via* one-way ANOVA with Tukey’s test.


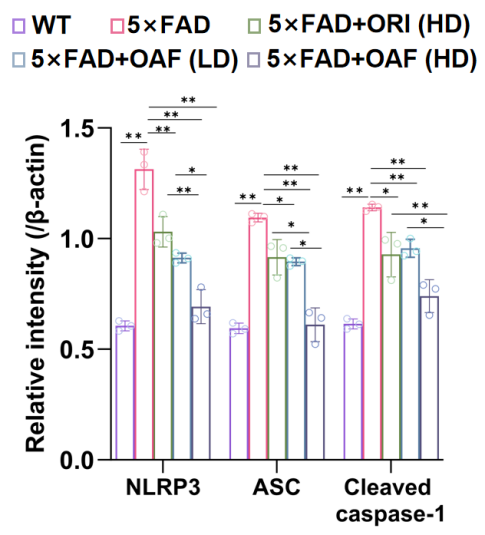


**FIGURE S32** The statistical analysis of the expression of NLRP3 inflammasome-related proteins in mice liver tissues. WT: wild-type. HD: 10 mg/kg (equivalent ORI dose). LD: 5mg/kg (equivalent ORI dose). Data were presented as mean ± SD, n=3. *^*^P* < 0.05, *^**^P*< 0.01. Statistical significance was calculated via one-way ANOVA with Tukey’s test.


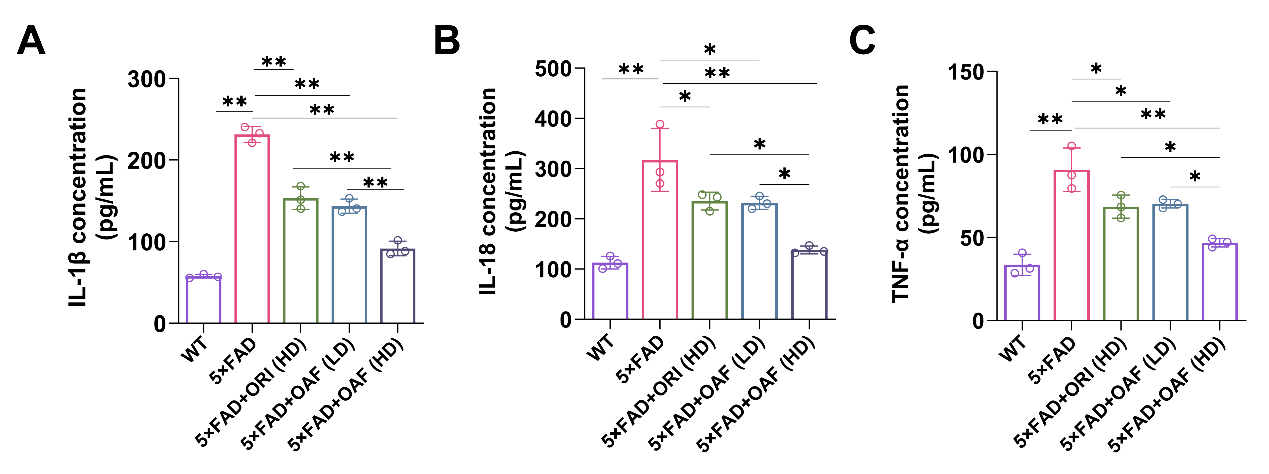


**FIGURE S33** OAF reduced the NLRP3-related inflammatory factors in 5×FAD mice. (A) IL-1β levels in mice serum. (B) IL-18 levels in mice serum. (C) TNF-α levels in mice serum. WT: wild-type. HD: 10 mg/kg (equivalent ORI dose). LD: 5mg/kg (equivalent ORI dose). Data were presented as mean ± SD, n=3. *^*^P* < 0.05, *^**^P*< 0.01. Statistical significance was calculated *via* one-way ANOVA with Tukey’s test.


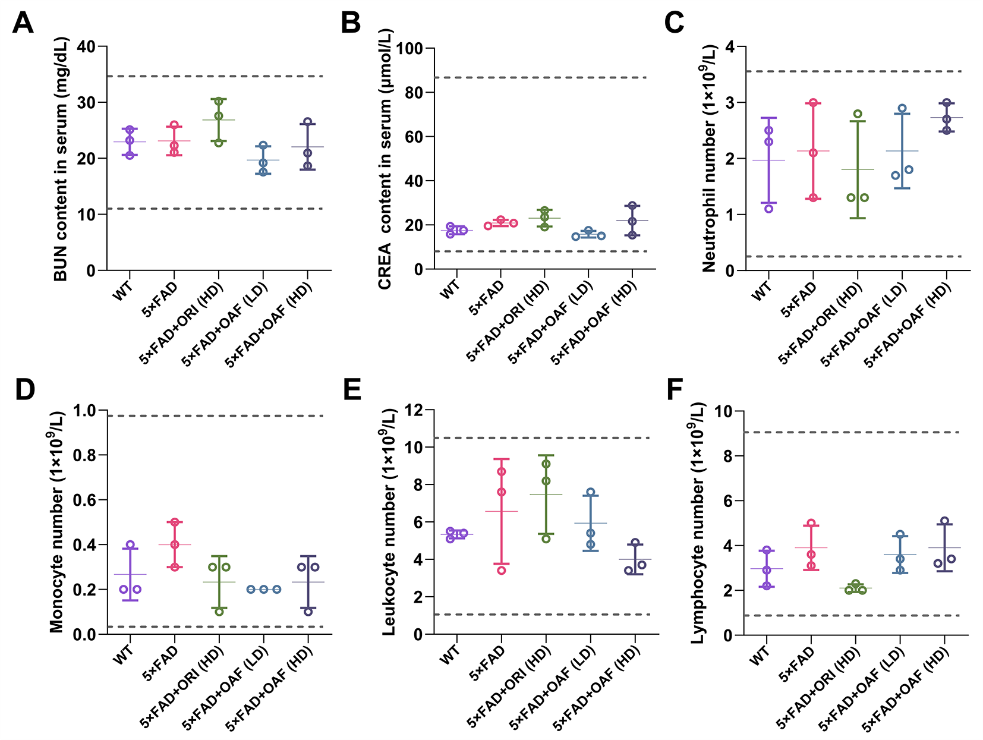


**FIGURE S34** The preliminary safety evaluation of ORI, OAF in 5×FAD mice. (A-B) The effect of OAF on renal function of 5×FAD mice. (C-F) The effect of OAF on immune cells in peripheral blood of 5×FAD mice. WT: wild-type. HD: 10 mg/kg (equivalent ORI dose). LD: 5mg/kg (equivalent ORI dose). Data were presented as mean ± SD, n=3. *^*^P* < 0.05, *^**^P*< 0.01. Statistical significance was calculated *via* one-way ANOVA with Tukey’s test.


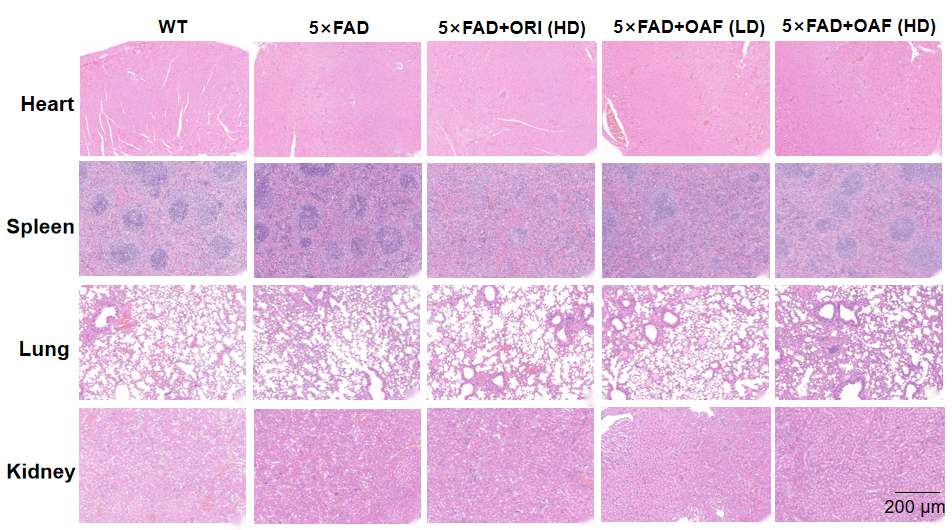


**FIGURE S35** H&E staining images of main organ tissues in WT and 5×FAD mice. WT: wild-type. HD: 10 mg/kg (equivalent ORI dose). LD: 5mg/kg (equivalent ORI dose).
